# Toxic Effects of Bis(4-hydroxyphenyl) Methane (BPF) on the Development and Reproduction of *Chironomus tentans*

**DOI:** 10.3390/jox15020041

**Published:** 2025-03-09

**Authors:** Chenglin Zhang, Zhen Wang, Huilin Liang, Shuai Sun, Weilong Xing, Bing Zhang, Feng Ge, Lei Wang

**Affiliations:** 1 Nanjing Institute of Environmental Science, Ministry of Ecology and Environment of the People’s Republic of China, Nanjing 210042, China; zhangchenglin@nies.org (C.Z.); wangzhen@nies.org (Z.W.); sunshuai@nies.org (S.S.); xingweilong@nies.org (W.X.); zhangbing@nies.org (B.Z.); 2 Key Laboratory of Pesticide Environmental Assessment and Pollution Control, Ministry of Ecology and Environment of the People’s Republic of China, Nanjing 210042, China; 3 School of Marine Science and Engineering, Nanjing Normal University, Nanjing 210023, China; 222612003@njnu.edu.cn

**Keywords:** chironomus tentans, Bis(4-hydroxyphenyl) methane, bisphenol F, reproductive toxicity, developmental toxicity

## Abstract

Bis(4-hydroxyphenyl) methane (BPF), as a bisphenolic compound, has toxic effects on organisms such as endocrine disruption and immobilization of growth and development. This study evaluated the effect concentrations of BPF on *Chironomus tentans* and investigated the impact of BPF exposure at various sub-lethal concentrations on the growth, development, and reproductive capacity of different instars of *C. tentans*. The results demonstrated that exposure at concentrations of 2.0, 2.5, 3.0, and 4.0 mg·L^−1^ delayed pupation, inhibited the development of imaginal discs, and caused an initial rise followed by a decline in the expression levels of genes related to larval development (*ecr*, *usp*, *e74*). Additionally, exposure at concentrations of 1.0, 1.5, and 2.0 mg·L^−1^ led to fluctuations in the expression levels of genes related to adult development and reproduction (*ecr*, *kr-h1*, *foxo*, *inr*, *pdk*, *akt*, and *vg*) in both female and male adults, with varying degrees of effect. Furthermore, BPF exposure inhibited male fertility, causing significant damage to the gonadal tissues, though it did not affect the final hatching of eggs. These findings indicate that BPF exhibits developmental and reproductive toxicity in *C. tentans*, with 2.0 mg·L^−1^ identified as the lowest effective concentration at which BPF affects pupation in midges.

## 1. Introduction

Bis(4-hydroxyphenyl) methane (BPF) is a bisphenol compound consisting of two phenol rings connected by a methylene bridge. BPF is widely used in food packaging, pipe linings, medical devices, coatings, plastics, and adhesives [1,2,3]. Following the restriction of bisphenol A (BPA) due to its reproductive toxicity, the production and use of BPF in industry have increased annually [4]. BPF is one of the most commonly used bisphenol A analogs (BPs) that does not evaporate from surface waters but can be transferred by sedimentation [5]. Although studies have shown that BPF in rivers and sediments can be completely degraded by microbial degradation and photochemical action [6,7], it has been detected in increasing amounts in various environmental matrices, such as surface water, groundwater [8,9], aquatic sediments [10,11], and soil [12]. Studies reported that 1890.51 ng·L^−1^ of BPF was detected in surface water [8], and the BPF content in sediments could reach 4.7 mg·L^−1^ [11].

Other BPs were initially developed as alternatives to BPA to mitigate its adverse effects on ecosystems. However, these alternatives did not have the desired effect as these BPs showed similar or even greater toxicity to aquatic organisms [13]. According to data released by the United States Environmental Protection Agency (USEPA) in 2015, BPF exhibits moderate acute aquatic toxicity (acute LC_50_ or EC_50_ > 10–100 mg·L^−1^) and high chronic aquatic toxicity (chronic LC_50_ or EC_50_ 0.1–1 mg·L^−1^) [5]. Studies have demonstrated that BPF poses significant threats to various niches in aquatic ecosystems. For instance, BPF at mg·L^−1^ can inhibit the bioluminescence of the marine bacterium *Vibrio fischeri* (Vibrionales: Vibrionaceae) [14], suppress the growth of the cyanobacterium *Synechococcus leopoliensis* (Synechococcales: Synechococcaceae) and the green alga *Desmodesmus subspicatus* (Sphaeropleales: Scenedesmaceae) [15,16], and induce oxidative stress and related gene upregulation in the rotifer *Brachionus koreanus* (Ploima: Brachionidae), leading to acute mortality [17]. Moreover, BPF can impair the mobility, reproduction, body length, and survival of *Daphnia magna* (Anomopoda: Daphniidae) [16,18], induce oxidative stress in zebrafish *Danio rerio* (Cypriniformes: Cyprinidae), cause cytotoxicity, disrupt lipid homeostasis, impair embryonic development, exhibit estrogenic effects, and even cause death [16,19,20,21,22]. Furthermore, BPF can interfere with the thyroid hormone (TH) signaling pathway in amphibians such as *Pelophylax nigromaculatus* (Anura: Ranidae) [23].

*Chironomus tentans* (Diptera: Chironomidae) belongs to the family Chironomidae, whose larvae is a benthic insect typically inhabit freshwater bodies, contributing to 70% to 80% of the total benthic biomass [24,25,26]. Both larvae and adults serve as essential food sources for higher trophic level organisms, thereby playing a crucial ecological role in the ecosystem [27]. Compared to *D. magna*, another aquatic model organism, *C. tentans* is more sensitive to certain environmental pollutants such as nonylphenol (NP), bisphenol A (BPA), and bis (2-ethylhexyl) phthalate (DEHP) [28]. Due to the high sensitivity to pollutants and short life cycle, *C. tentans* larvae are frequently used as bioindicators and for toxicological assessments, making them a standard test organism [29,30,31,32]. Studies have shown that *C. tentans* is more sensitive to BPAF and BPF than *Marisa cornuarietis* (Architaenioglossa: Ampullariidae) and *Scenedesmus obliquus* (Sphaeropleales: Scenedesmaceae) [33]. Exposure to BPF induces the upregulation of gene expression, such as *ecr* and *e74*, in the salivary gland cells of *C. tentans* larvae and alters the processes of embryonic hatching and larval development [34].

Similar physico-chemical properties of different pollutants often indicate similar environmental fates. The logarithm of the octanol–water partition coefficient (K_ow_) is a measure of a substance’s hydrophilicity and tendency to dissolve in water. The log K_ow_ of BPA is 3.64, while those of BPF and most BPs are greater than 3, indicating similar lipophilicity. Additionally, log K_ow_ is positively correlated with the logarithm of the bioconcentration factor (BCF), which reflects the bioaccumulation potential of a substance [35]. Lipophilicity is the primary driving force for the accumulation of BPs in aquatic organisms, and the higher log K_ow_ causes BPA and its alternatives to be more concentrated in sediments rather than in water or soil [36]. Although existing studies have demonstrated the negative impacts of BPF on aquatic organisms, including mutagenic and genotoxic effects, developmental toxicity, and oxidative toxicity, these findings remain limited [13,37]. Therefore, it is essential and practical to assess the effects of BPF on organisms in sediments. As a holometabolous insect, *C. tentans* larvae, from the first- to fourth-instar stages, live in the sediments at the bottom of water bodies [38]. This unique habitat makes *C. tentans* an ideal model for evaluating the effects of BPs on aquatic organisms. Therefore, this study evaluated the effects of BPF on *C. tentans*, including acute immobilization, chronic lethality, developmental toxicity, and reproductive toxicity, with the aim of providing a more comprehensive assessment of the impact of BPs on aquatic organisms.

## 2. Materials and Methods

### 2.1. Experimental Materials

The *C. tentans* used in the experiments were continuously cultured in the laboratory for over 10 generations. Dechlorinated freshwater, aerated for more than 48 h, was used for cultivation, with gentle aeration maintained in the culture tanks. The temperature was controlled at 23 ± 2 °C, with a light-dark cycle of 16:8 h and a light intensity of 800 ± 200 lux. During the feeding period, artificial feed (Longxing, Qingdao, China) was provided three times a week at a rate of 1.0 mg per worm per feeding. The BPF (purity > 99.5%, Solarbio, Beijing, China) used in this study was dissolved in DMSO to prepare the stock solution, with the DMSO final concentration less than 0.01% (*v*/*v*). with different doses added based on the required concentrations.

### 2.2. Exposure Design and Sample Collection

The experiment was composed of three parts.

1. Acute toxicity test. Eight concentrations of BPF (0.1, 0.5, 1.0, 2.0, 4.0, 8.0, 10.0, and 20.0 mg·L^−1^) were tested, with four replicates for each concentration. Each group contained five first-instar larvae. The exposure period lasted 48 h, during which no feeding or aeration was provided. Larval immobilization was recorded at 24 and 48 h, and the relevant rate was calculated. Larvae were considered immobilized if they did not exhibit movement within 15 s after being gently stimulated with water using a Pasteur pipette.
2. Developmental toxicity test. Before the experiment, egg masses produced within 8 h were cultured until the early fourth-instar stage, at which point the larvae were used as experimental subjects. The larvae were randomly added to 50.0 mL BPF solutions at various concentrations and a control group without it. The concentrations were set according to the acute toxicity test, specifically concentrations near EC_10_, EC_20_, and EC_50_ (1.5, 2.0, 2.5, 3.0, and 4.0 mg·L^−1^). Each group consisted of three replicates, with five larvae per replicate. Experimental conditions were consistent with Section 2.1, with feeding at a rate of 1.0 mg per larva per day and gentle aeration maintained throughout the experiment. Larval survival and pupation were recorded every 24 h. The experiment continued until all larvae either pupated or died, with dead larvae removed daily. Additional replicate groups were established for the control, 2.0, and 4.0 mg·L^−1^ concentrations to collect samples for histopathological analysis of the imaginal discs. Development-related gene expression in the control and 2.0 mg·L^−1^ groups was also measured at 0, 72, 120, and 144 h.
3. Reproductive toxicity test. Three exposure concentrations were set, with the highest concentration based on the lowest effective concentration from the developmental toxicity test, specifically 0.5, 1.0, and 2.0 mg·L^−1^ groups, along with a 0 mg·L^−1^ control group. Each group consisted of three replicates, with 20 fourth-instar larvae per replicate. Experimental conditions were consistent with the developmental toxicity test. During the exposure period, the emergence of *C. tentans* in the control and treatment groups was monitored. Newly emerged adults were collected at 08:00 and 20:00 daily and transferred by sex to new 5 L beakers containing aerated freshwater, with a 1:1 sex ratio for each treatment to assess the reproductive capacity of treated females and males. Newly produced egg masses were transferred to 6-well plates containing aerated water, and hatching was observed under a microscope every 24 h. The spawning rate was calculated as the total number of egg masses per experimental container divided by the total number of live females in it. Additional replicate groups were established in the control and treatment groups to collect samples for histopathological analysis of the reproductive system in newly emerged adults, less than 12 h old. The expression levels of ecdysone, juvenile hormone, and insulin pathway-related genes in newly emerged adults less than 12 h old were also measured.

To confirm the stability of BPF in the test solutions, additional replicate groups were established. The concentration of BPF in the solutions from 0 to 48 h was measured using liquid chromatography–high–resolution mass spectrometry (Orbitrap Exploris 240, Thermo Fisher Scientific, Waltham, MA, USA). The chemical analysis results (Table S1) indicated that BPF concentration variations were within 20% of the initial concentration. Therefore, results were expressed in terms of the prepared concentration. All solutions were renewed every 48 h during the tests.

### 2.3. Histopathological Analysis

In the developmental toxicity test, larvae from the control, 2.0, and 4.0 mg·L^−1^ groups were selected for analysis. Serial paraffin sections and hematoxylin-eosin (HE) staining were performed on the larvae’s second to fourth abdominal segments (cross-sections). Each sample was sectioned 10 times at 10 µm intervals to assess the impact of BPF on larval development. Paraffin sections with intact tissue and clear staining were selected to measure the maximum cross-sectional area of the imaginal discs in at least five larvae from each group.

In the reproductive toxicity test, three adults of each sex, which had emerged within 12 h, were randomly selected from each of the four treatment groups. These adults were fixed in 4% paraformaldehyde, and serial paraffin sections and HE staining were performed on their abdomens (longitudinal sections). Each sample was continuously sectioned 10 times at 10 µm intervals to evaluate the impact of BPF exposure on the reproductive system development in male and female adults.

Additionally, egg masses from the control and 2.0 mg·L^−1^ groups in the reproductive toxicity test were observed under a microscope.

### 2.4. mRNA Expression Levels of Larvae and Adults

Total RNA from each sample was extracted using the Trizol method, with the RNA quality checked according to the OD_260/280_ ratio. Agarose gel electrophoresis was used to evaluate the integrity of the extracted RNA and the extent of DNA contamination. Once the RNA quality met the required standards, reverse transcription was performed using the Hifair III 1st Strand cDNA Synthesis SuperMix for qPCR (gDNA digester plus) kit to synthesize cDNA. Real-time quantitative PCR (qRT-PCR) was conducted using the SYBR GREEN dye method to analyze the expression of ecdysone, juvenile hormone, and insulin-related genes in *C. tentans*. The reference used for normalization was the GAPDH gene, and the specific genes analyzed included *Ecdysone receptor* (*ecr*), *Ultraspiracle* (*usp*), *E26 transformation-specific* (*e74*), *Krüppel-homolog 1* (*kr-h1*), *Forkhead box* (*foxo*), *Insulin Receptor* (*inr*), *Phosphoinositide-dependent kinases* (*pdk*), *Serine/threonine kinases* (*akt*), and *Vitellogenin* (*vg*). The genes in Table S2 were subjected to qRT-PCR using the Genious 2×SYBR Green Fast qPCR Mix kit. Detailed experimental manipulations are described in the Supplementary Material. Relative quantification of gene expression was performed using the 2^−ΔΔCt^ method [34].

### 2.5. Statistical Analysis

Immobilization rates were statistically analyzed using SPSS 26.0. EC_10_, EC_20_, and EC_50_ values were calculated through probit regression analysis using the LOGIT link function. The relative quantification of RT-qPCR data was performed using the 2^−ΔΔCt^ method, where the relative expression levels of each gene at different developmental stages were normalized against the gene expression levels of successfully emerged male and female adults in the control group. All the data were expressed as mean ± SEM. Data from each group were analyzed using one-way analysis of variance (ANOVA), followed by the least significant difference (LSD) test to assess the significance of differences within and between groups. A *p*-value of less than 0.05 was considered statistically significant, and different letters were used to denote significant differences. All gene expression data were reported as mean ± standard deviation.

## 3. Results

### 3.1. Acute Toxicity of BPF to Larvae

**Figure 1:**
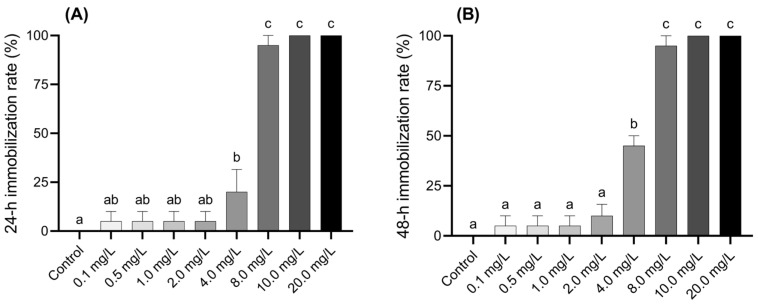
Acute effects of BPF on *Chironomus tentans* larvae. (**A**) 24 h immobilization rate; (**B**) 48 h immobilization rate. Sample size (*n*) = 5. The same letter indicates statistical insignificance (*p* > 0.05), while different letters indicate statistical significance (*p* < 0.05).

The inhibition of larval activity at 24 and 48 h under various concentrations of BPF is shown in Figure 1A,B. Compared to the control group, larvae exposed to 4.0 mg·L^−1^ BPF showed a 20% inhibition rate after 24 h, which increased to 45% after 48 h, indicating a significant obstruction of larval activity (*p* < 0.05). Larvae exposed to concentrations above 8.0 mg·L^−1^ exhibited a significant inhibition rate of over 95% after 24 h or more of exposure (*p* < 0.05). The inhibition rate of the larvae was positively correlated with both BPF exposure time and concentration. The effective concentration was positively correlated with the inhibition rate and negatively correlated with exposure time (Figure 1 and Table S4).

### 3.2. Developmental Toxicity of BPF

#### 3.2.1. Changes in Physiological Indexes Related to Pupation

**Figure 2:**
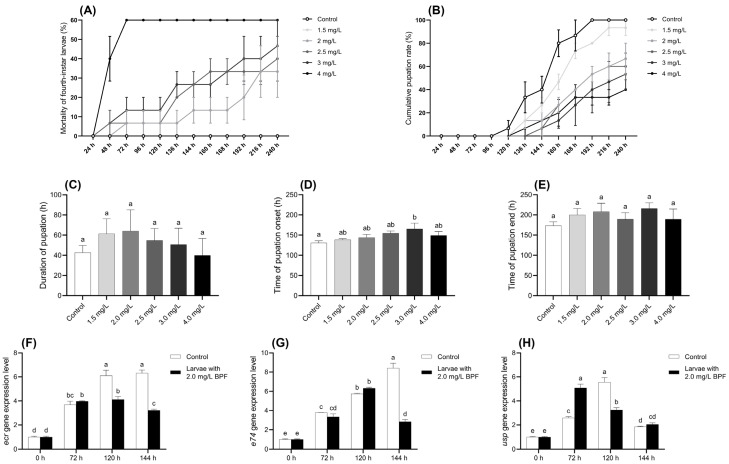
Effects of BPF on physiological indexes related to pupation of *C. tentans* larvae. (**A**) Chronic mortality of fourth-instar larvae; (**B**) cumulative pupation rate; (**C**) duration of pupation; (**D**) average time of pupation onset; (**E**) average time of pupation end. Changes at development-related gene expression levels in *C. tentans* larvae. (**F**) *ecr*; (**G**) *usp*; (**H**) *e74*. Sample size (*n*) = 5 for Figure 2A–E, and *n* = 3 for Figure 2F–H. The same letter indicates statistical insignificance (*p* > 0.05), while different letters indicate statistical significance (*p* < 0.05).

During the exposure period, all larvae in the control group survived and developed normally to the pupation stage. In contrast, the mortality rate of larvae in the BPF-treated groups was positively correlated with the exposure concentration. When the BPF concentration was below 1.5 mg·L^−1^, the mortality rate of fourth-instar larvae at all time points did not differ significantly from the control group (*p* > 0.05). However, at concentrations of 2.0, 2.5, 3.0, and 4.0 mg·L^−1^, the mortality rates after 216, 136, 136, and 48 h, respectively, were significantly higher than those of the control group (*p* < 0.05) (Figure 2A and Table S4).

The cumulative pupation rate of larvae exposed to BPF are presented in Figure 2B. Compared to the control group, BPF exposure delayed larval pupation, and the cumulative pupation rate decreased with increasing BPF concentration. Although a decreasing trend in the cumulative pupation rate was observed at BPF concentrations within 1.5 mg·L^−1^, it was not statistically significant (*p* > 0.05) except at 160 h. However, when the BPF concentration exceeded 2 mg·L^−1^, pupation was delayed in the treatment groups. At 240 h, the pupation rates in these treatment groups of 2–4 mg·L^−1^ were significantly lower than those in the control group (*p* < 0.05) (Table S5).

The effects of BPF on pupation time and the duration of various developmental stages are shown in Figure 2C–E. Compared to the control group, exposure to 3.0 mg·L^−1^ BPF significantly delayed the average initiation of pupation (*p* < 0.05), with a delayed trend also observed in the other exposure groups (*p* > 0.05). Despite these delays, BPF exposure did not result in statistically significant changes in the average pupation time or the average completion time of pupation, although these parameters exhibited fluctuations across different concentrations (*p* > 0.05).

#### 3.2.2. Changes in Expression Levels of Development-Related Genes

Analysis of the developmental genes in *C. tentans* larvae revealed distinct expression patterns under BPF exposure. In the control group, the expression level of the gene *ecr* gradually increased across the three time points, peaking at 144 h. However, under 2.0 mg·L^−1^ BPF exposure, the *ecr* expression peaked earlier at 120 h and began to decline by 144 h, indicating a disruption in its normal expression pattern compared to the control group. Gene *e74* expression peaked at 120 h under BPF exposure and began to decrease by 144 h. For the gene *usp*, expression under 2.0 mg·L^−1^ BPF exposure reached its peak at 72 h and then started to decline at 120 h, suggesting that BPF accelerates the gene expression but also shortens its duration of peak activity as exposure time increases. The shifts in peak expression times for these genes under BPF exposure indicate that BPF interfered with the normal developmental processes of *C. tentans* larvae (Figure 2F–H).

#### 3.2.3. Imaginal Disc Histopathology

**Figure 3:**
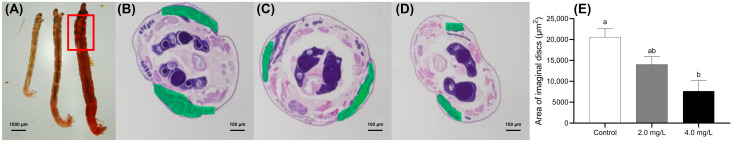
Effects of BPF on the development of imaginal discs in *C. tentans*. (**A**) Overall view of larvae; morphological changes in imaginal discs: (**B**) control group; (**C**) 2 mg·L^−1^ BPF group; (**D**) 4 mg·L^−1^ BPF group; (**E**) average area of imaginal discs. The green section represents the area of the imaginal discs. Sample size (*n*) = 6. The same letter indicates statistical insignificance (*p* > 0.05), while different letters indicate statistical significance (*p* < 0.05).

The inhibition of imaginal disc development was more pronounced in the 4.0 mg·L^−1^ group than in the 2.0 mg·L^−1^ group, indicating a dose-dependent adverse effect of BPF on the development of these structures (Figure 3). To investigate the potential impact of BPF exposure on larval development, the location of the imaginal discs in fourth-instar *C. tentans* larvae was identified (marked in green) according to the identification in *Aedes aegypti* (Diptera: Culicidae) [39].

In the serial paraffin sections, Figure 3B–D correspond to the maximum areas of the imaginal discs in the control, 2.0, and 4.0 mg·L^−1^ groups, respectively. The development of the imaginal discs was quantified by measuring their area in the serial sections. Compared to the control group, the imaginal disc area in the 4.0 mg·L^−1^-treated group was significantly shrunken (*p* < 0.05) (Figure 3E).

### 3.3. Effects of BPF on Reproduction Function

#### 3.3.1. Changes in Reproductive Organ Morphology

**Figure 4:**
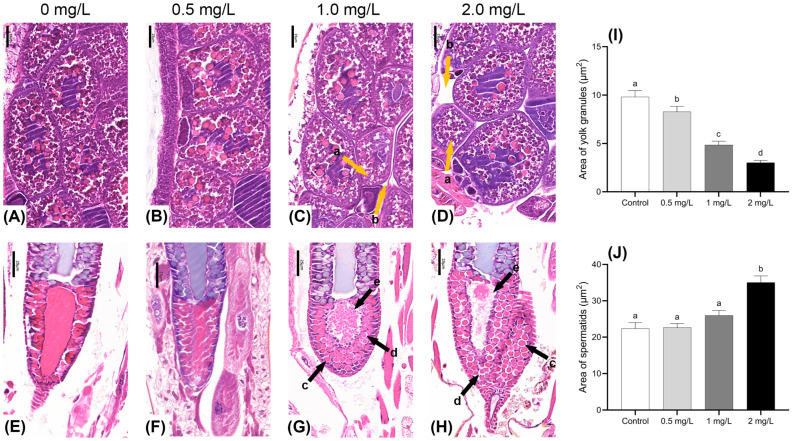
Effects of BPF on gonadal development in *C. tentans.* Female: (**A**): control group; (**B**) 0.5 mg·L^−1^ BPF group; (**C**) 1.0 mg·L^−1^ BPF group; (**D**) 2.0 mg·L^−1^ BPF group; male: (**E**): control group; (**F**) 0.5 mg·L^−1^ BPF group; (**G**) 1.0 mg·L^−1^ BPF group; (**H**) 2.0 mg·L^−1^ BPF group. (**I**) The area of the yolk granules; (**J**) the area of the spermatids. Arrow: a: shrunken yolk granules; b: enlarged intercellular space between yolk granules; c: enlarged volume and changed morphology of spermatids; d: enlarged intercellular space between spermatids; e: ruptures of mature spermatids. Sample size (*n*) = 30. The same letter indicates statistical insignificance (*p* > 0.05), while different letters indicate statistical significance (*p* < 0.05).

The study examined the effects of BPF exposure on the gonadal development of adult males and females at varying concentrations. Morphological changes in the ova and spermatids were observed using HE-stained paraffin sections. Compared to the control group, ovum tissues in the treatment groups displayed a noticeable reduction in the volume of yolk granules, and a higher number of immature cells were present. The intercellular space between yolk granules was simultaneously enlarged (Figure 4A–D). The area of the yolk granules region was significantly reduced at all concentrations (*p* < 0.05) (Figure 4I). In males exposed to 1.0 and 2.0 mg·L^−1^ BPF, significant alterations were observed in the seminal vesicle. There was an enlargement of the spermatid on the side near the seminal duct along with morphological change and increased intercellular gaps. Furthermore, mature spermatids near the seminal duct exhibited signs of rupture, with these ruptured ones appearing dark purple (Figure 4G,H). The area of the spermatid’s region showed an increasing trend and was significantly larger in the 2 mg·L^−1^ concentration group (*p* < 0.05) (Figure 4J).

#### 3.3.2. Changes in Spawning Rate

**Figure 5:**
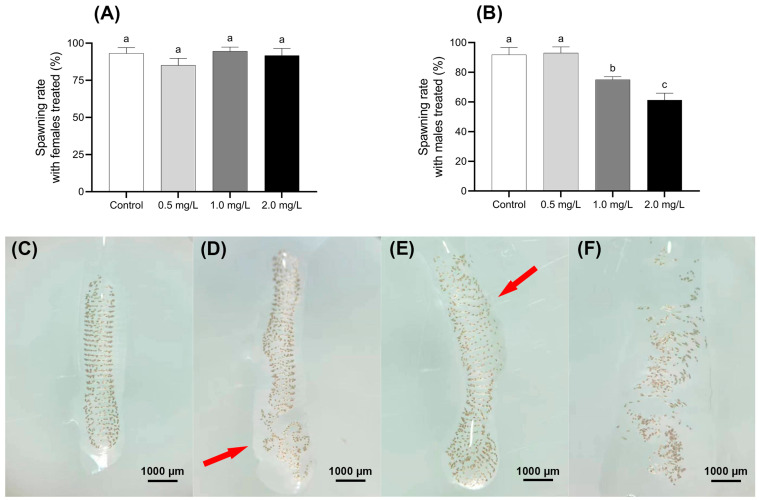
Effect of BPF on oviposition of *C. tentans*. Effects of BPF exposure on the spawning rate of different sexes of *C. tentans*. (**A**) Treated females × untreated males; (**B**) treated males × untreated females; appearance of egg tissue: (**C**) in the control group; (**D**) 0.5 mg·L^−1^ BPF group; (**E**) 1 mg·L^−1^ BPF group; (**F**) 2 mg·L^−1^ BPF group. Arrow: the loss of intact shape, disruption of the spiral arrangement, thinning of the gelatinous layer, and the absence of uniform wrapping. Sample size (*n*) = 20. The same letter indicates statistical insignificance (*p* > 0.05), while different letters indicate statistical significance (*p* < 0.05).

The impact of BPF on the spawning rate of *C. tentans* under different treatment is shown in Figure 5A,B. When treated females mated with untreated males, there was no significant difference in the spawning rates across the four BPF-treated groups, with rates ranging from 85% to 95% compared to the control group (*p* > 0.05). However, a different trend was observed in the groups where treated males were paired with untreated females. The spawning rate in the 0.5 mg·L^−1^ male-treated group was 93%, which did not significantly differ from the control group (*p* > 0.05). In contrast, the spawning rates in the 1.0 and 2.0 mg·L^−1^ male-treated groups were significantly lower, at 75% and 61%, respectively (*p* < 0.01).

#### 3.3.3. Changes in Egg Mass Morphology

In the control group, the egg masses of *C. tentans* were well-structured, with uniformly arranged egg individuals and no visible damage to the egg grains. In contrast, the egg masses from groups where males were exposed to 1.0 and 2.0 mg·L^−1^ BPF showed notable deformation and uneven egg arrangement. Additionally, the outer gelatinous layer of these masses was often sparse and not uniformly wrapped around the egg grains. These structural changes resulted in the egg masses losing their intact shape, and the spiral arrangement of the egg grains becoming disordered. Despite these structural changes, the developmental time consumption of most eggs in the treated groups was similar to that of the control group (Figure 5C–F).

#### 3.3.4. Changes in Gene Expression Levels Related to Hormone Secretion and Energy Metabolism

**Figure 6:**
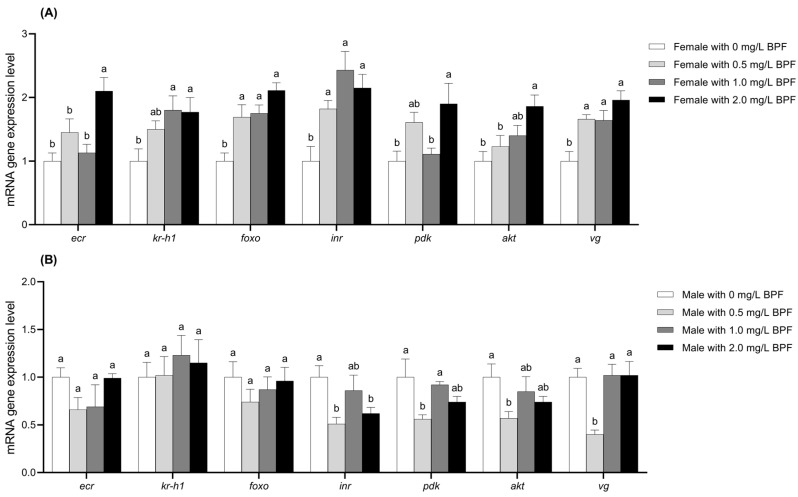
Effects of BPF on the expression levels of *ecr*, *kr-h1*, *foxo*, *inr*, *pdk*, *akt*, and *vg* genes in *C. tentans* adults. (**A**) Females; (**B**) males. Sample size (*n*) = 6. The same letter indicates statistical insignificance (*p* > 0.05), while different letters indicate statistical significance (*p* < 0.05).

Compared to the control group, the expression levels of the genes *foxo*, *inr*, and *vg* in female *C. tentans* were significantly upregulated across all three treated groups (*p* < 0.01). The genes *ecr*, *pdk*, and *akt* were significantly upregulated in the 2.0 mg·L^−1^ treatment group (*p* < 0.01) and showed an upward trend in the 0.5 and 1.0 mg·L^−1^ treatment groups, although the differences were not statistically significant (*p* > 0.05). The expression level of *kr-h1* was significantly upregulated in the 1.0 and 2.0 mg·L^−1^ groups (*p* < 0.01), with an upward trend observed in the 0.5 mg·L^−1^ group, though this difference was not significant (*p* > 0.05) (Figure 6A).

For treated males, the expression levels of *ecr* and *foxo* exhibited a downward trend across all BPF concentrations, although these differences were not statistically significant compared to the control group (*p* > 0.05). The gene *inr* expression was significantly reduced in the 0.5 and 2.0 mg·L^−1^ treatment groups (*p* < 0.01), with a slight downward trend in the 1.0 mg·L^−1^ treatment group (*p* > 0.05). The expression levels of the *pdk* and *akt* genes were significantly downregulated in the 0.5 mg·L^−1^ group (*p* < 0.01), with a slight downward trend in the 1.0 and 2.0 mg·L^−1^ groups (*p* > 0.05). The *vg* gene expression was significantly downregulated in the 0.5 mg·L^−1^ group (*p* < 0.01), while it showed an upward trend in the 1.0 and 2.0 mg·L^−1^ groups without significant differences (*p* > 0.05). *kr-h1* gene expression showed an upward trend across all three treatment groups, although not significant (*p* > 0.05) (Figure 6B).

## 4. Discussion

To mitigate adverse ecological impacts, BPs were introduced as alternatives to BPA. However, the effectiveness of this approach has not met expectations. In particular, BPF has been identified in a number of aquatic environments across Asia, with concentrations exceeding 1 mg·L^−1^ recorded at multiple sampling points in Japan’s Tamagawa River, South Korea’s Han River, and China’s Pearl River [40]. BPs exhibit toxic effects on aquatic organisms that are comparable to, or even more severe than, those of BPA [35]. Specifically, the 48 h EC_50_ values for BPF are 2300 µg·L^−1^ for *C. tentans* larvae and 8700 µg·L^−1^ for *D. magna*; the 96 h LC_50_ values are 8000 µg·L^−1^ for *M. cornuarietis* and 9510 µg·L^−1^ for *D. rerio* [41]. In comparison, BPA’s 96 h EC_50_ values are 2700 µg·L^−1^ for *C. tentans* larvae and 8629 µg·L^−1^ for *D. magna* [33]. The 96 h LC_50_ values for BPA are 2240 µg·L^−1^ for *M. cornuarietis* and 6930 µg·L^−1^ for *D. rerio* [41,42]. The current study found a similar 48 h EC_50_ of 3.44 mg·L^−1^ for *C. tentans* exposed to BPF. Notably, a BPF concentration of 4.0 mg·L^−1^ significantly immobilizes larvae, with immobilization rates exceeding 95% at concentrations above 8.0 mg·L^−1^, indicating pronounced acute toxicity effects on *C. tentans*. Given its relatively low and stable EC_50_ values, *C. tentans* is used as an indicator for evaluating the acute effects of BPs.

Pupation and eclosion are critical physiological processes for holometabolous insects and impact population development and continuity. Research has shown that BPA at a concentration as low as 1 µg·L^−1^, can induce early eclosion and reduce eclosion rates in *C. riparius* [43]. Accordingly, the developmental toxicity of various BPF concentrations was evaluated based on the EC_50_ for *C. tentans* larvae. Pupation, as one of the eclosion parameters, is commonly used as developmental indicators [44]. Imaginal discs, composed of undifferentiated cells, are unique structures during the larval stage of insects, developing into wings and other structures such as the notum. The development of these structures is crucial for successful pupation and eclosion [45]. In this study, the cumulative pupation rate of *C. tentans* larvae decreased with increasing BPF concentration, which may be related to BPF-induced mortality in late-instar larvae before pupation. Furthermore, exposure to BPF resulted in delays in pupation and eclosion, as well as impacts on the development of imaginal discs, ultimately leading to developmental delays. This is reflected in the lag in the average start time of pupation and variability in pupation start and end times among the groups. Previous studies have also reported developmental toxicity of BPF, with exposure to BPA over two generations leading to varying degrees of delayed eclosion in both female and male *C. riparius*. Furthermore, a 20 mg·L^−1^ concentration of BPF can induce developmental abnormalities such as craniofacial abnormality, spinal malformation, cranial hemorrhage, and yolk sac deformity in zebrafish larvae [21]. These findings suggest that the toxic effects and mechanisms of BPs are similar to those of BPA, indicating that BPs may not be a so-called sound alternative. As the production and application of BPs increase, it is imperative that their negative biological effects, including developmental toxicity, are given due consideration.

Reproduction represents a further vital physiological process for the maintenance of population continuity, with the gonads constituting a principal target organ for environmental pollutants in the larvae of *C. tentans*. This study provides new insights into the microscopic structure of the reproductive systems in both female and male *C. tentans* adults, highlighting BPF-induced reproductive toxicity from a tissue damage perspective. The findings suggest that BPF exposure did not significantly impact the reproductive capability of female *C. tentans*, but it may reduce the reproductive capability of males. While no significant tissue damage was observed in the ova of female adults across all experimental groups, spermatid in the males exposed to 1.0 and 2.0 mg·L^−1^ BPF exhibited signs of rupture. This implies that BPF disrupts the normal development and maturation of male *C. tentans* reproductive cells, potentially affecting the proper production and hatching of fertilized eggs and leading to a decreased spawning rate. Despite the absence of documented evidence pertaining to the sex-specific impact of BPs on *C. tentans*, there is a growing body of research indicating that BPA may potentially induce feminisation in aquatic organisms [46]. It is plausible that BPF’s reproductive toxicity might also involve the regulation of reproductive genes. Moreover, BPF toxicity may have genetic implications, as evidenced by studies involving HepG2 cell lines [47], chicken DT40 cell lines [48], and MCF-7 cell lines [49]. Matthew et al. reported significant hatching failures in *C. tentans* egg masses exposed to 10.4 mg·L^−1^ BPF [50]. In this study, BPF disrupted the macroscopic structural integrity of egg masses, though no significant damage to hatching or developmental performance was observed. This may be attributed to the relatively low concentrations of BPF used.

To gain further insight into the mechanism of BPF toxicity, we analyzed the expression levels of key genes involved in *C. tentans* development and reproduction. During the process of early development, the larvae undergo several molts before reaching final metamorphosis; the expression levels of molting-related genes reflect their growth. Ecdysone (Ecd) promotes the binding of ecdysone receptors (ECR) and ultraspiracle proteins (USP) to form heterodimers, which activate downstream genes such as *e74* to facilitate molting. Previous studies have indicated that Bisphenol P (BPP) exposure can upregulate the expression levels of *ecr*, *usp*, and *e74* genes in *C. tentans* after 3 h [34]. In this study, the expression levels of *ecr*, *usp*, and *e74* in female larvae initially increased but then decreased during exposure, with *ecr* and *usp* showing more pronounced changes than *e74*. It is speculated that BPF interferes with the normal functioning of the ecdysone signaling pathway in *C. tentans* larvae, potentially disrupting the binding of ECR and USP to form heterodimers and affecting the expression of downstream genes such as *e74*.

Ecdysteroids and juvenile hormones (JH) play crucial roles in regulating insect tissue apoptosis and developmental physiology by modulating downstream genes such as *ecr* and *kr-h1*. Cytokines activate the PI3K/AKT signaling pathway, leading to the phosphorylation of AKT, which regulates physiological activities, embryonic development, and immune responses [51,52]. PDK-1 modulates the activity of this pathway. Vitellogenin, which is associated with embryonic development and energy supply, is a precursor to yolk proteins that include carotenoids, carbohydrates, lipids, and proteins. Vitellogenin is frequently employed as a marker for ovarian development in crustaceans [53]. The *foxo* gene plays a pivotal role in regulating growth, cell differentiation, metabolism, immunity, and apoptosis., In particular, *foxo3* regulates and inhibits primordial follicle activation. Its expression decreases significantly when female ovaries are stimulated by estrogen and gonadotropins [54]. The *foxo3* gene is involved in embryogenesis, feeding regulation, and glucose metabolism in various organisms [55]. It is a large phosphoglycoprotein found in the blood of female animals, providing essential energy reserves for embryonic development [56]. In this study, BPF exposure showed sex-specific effects in *C. tentans*. BPF generally resulted in significant upregulation of gene expression in females, while in males, the majority of genes expression levels decreased except for *kr-h1*. It is inferred that BPF induces stress in *C. tentans* adults, prompting an elevation in energy metabolism in females to counteract this stress. Consequently, genes related to energy metabolism, such as *vg* and *foxo*, are upregulated in females. The upregulation of *ecr*, *kr-h1*, *pdk*, and *akt* might result from the allocation of energy towards coping with BPF stress, leaving insufficient energy to maintain hormonal homeostasis. Such an imbalance could induce the upregulation of these genes. Given that *vg* levels are higher in females, the observed downregulation of *vg* and other genes in this study may indicate that BPF stress exceeds the threshold of tolerance in males, leading to imbalanced energy metabolism and hormonal secretion disorders, ultimately suppressing gene expression. Additionally, the discrepancies in gene expression patterns between the sexes may be attributable to tissue morphological damage. BPF-induced damage in male reproductive organs could contribute to the observed disruptions in hormonal secretion and energy metabolism.

## 5. Conclusions

This study revealed that BPF exposure not only has acute toxic effects on *C. tentans* (48 h EC_50_ 3.44 mg·L^−1^), but also has negative effects on development and reproduction. In terms of development, BPF can lead to a decrease in the pupation rate and a delay in larval development, and this effect can be produced at a concentration of 2.0 mg·L^−1^. In terms of reproduction, BPF exposure can damage the integrity of male adult germ cells, leading to a decrease in egg laying rate and changes in egg mass morphology. This may be because BPF affects the expression levels of genes in the hormone signaling pathway that controls growth and energy metabolism in the body, thereby affecting early development and reproductive processes.

## Data Availability

The original contributions presented in this study are included in the article/Supplementary Material. Further inquiries can be directed to the corresponding author.

## Supplementary Materials

The following supporting information can be downloaded at: https://www.mdpi.com/article/10.3390/jox15020041/s1, Table S1: ANOVA results of acute toxic test.; Table S2: Post hoc tests results of 24-h acute toxicity test; Table S3: Post hoc tests results of 48-h acute toxicity test; Table S4: Chi-square test result for acute toxic test; Table S5: Confidence limits of probit analysis (24 h); Table S6: Confidence limits of probit analysis (48 h); Table S7: ANOVA results of fourth-instar larvae mortality rate; Table S8: Post hoc tests results of fourth-instar larvae mortality rate; Table S9: ANOVA results of cumulative pupation rate; Table S10: Post hoc tests results of cumulative pupation rate; Table S11: ANOVA results of duration of pupation, time of pupation onset, and time of pupation end; Table S12: Post hoc tests results of duration of pupation, time of pupation onset, and time of pupation end; Table S13: ANOVA results of gene expression levels of *ecr*, *e74*, and *usp*; Table S14: Post hoc tests results of gene expression levels of *ecr*, *e74*, and *usp;* Table S15: ANOVA results of area of imaginal discs; Table S16: Post hoc tests results of area of imaginal discs; Table S17: ANOVA results of area of yolk granules; Table S18: Post hoc tests results of area of yolk granules; Table S19: ANOVA results of area of spermatids; Table S20: Post hoc tests results of area of spermatids; Table S21: ANOVA results of spawning rate of treated females × untreated males; Table S22: Post hoc tests results of spawning rate of treated females × untreated males; Table S23: ANOVA results of spawning rate of treated males × untreated females; Table S24: Post hoc tests results of spawning rate of treated males × untreated females; Table S25: ANOVA results of gene expression levels of *ecr*, *kr-h1*, *foxo*, *inr*, *pdk*, *akt*, and *vg* in females; Table S26: Post hoc tests results of gene expression levels of *ecr*, *kr-h1*, *foxo*, *inr*, *pdk*, *akt*, and *vg* in females; Table S27: ANOVA results of gene expression levels of *ecr*, *kr-h1*, *foxo*, *inr*, *pdk*, *akt*, and *vg* in males; Table S28: Post hoc tests results of gene expression levels of *ecr*, *kr-h1*, *foxo*, *inr*, *pdk*, *akt*, and *vg* in males.
