# Supplementary material for: Toxic Effects of Bis(4-hydroxyphenyl) Methane (BPF) on the Development and Reproduction of Chironomus tentans
Source: J Xenobiot. 2025 Mar 9;15(2):41. doi: 10.3390/jox15020041 (PMC11932282; doi:10.3390/jox15020041)
Supplement: Supplementary file 1 [file jox-15-00041-s001.zip › jox-3362758-supplementary.pdf]

Journal of Xenobiotics

MDPI

Supplementary Materials: Toxic Effects of Bis(4-hydroxyphenyl) Methane (BPF) on the Development and Reproduction of *Chironomus tentans*

Table S1. ANOVA results of acute toxic test.

		Sum of Squares	df	Mean Square	F	Sig.
24-h	Between Groups	68222.222	8	8527.778	74.274	0
	Within Groups	3100	27	114.815		
	Total	71322.222	35			
48-h	Between Groups	68222.222	8	8527.778	74.274	0
	Within Groups	3100	27	114.815		
	Total	71322.222	35			

Table S2. Post hoc tests results of 24-h acute toxicity test.

Multiple Comparisons							
Dependent Variable: Mortality							
	(I) Concentration	(J) Concentration	Mean Difference (I-J)	Std. Error	Sig.	95% Confidence Interval	
						Lower Bound	Upper Bound
LSD	0	0.1	-5	7.57677	0.515	-20.5462	10.5462
		0.5	-5	7.57677	0.515	-20.5462	10.5462
		1	-5	7.57677	0.515	-20.5462	10.5462
		2	-5	7.57677	0.515	-20.5462	10.5462
		4	-20.00000*	7.57677	0.014	-35.5462	-4.4538
		8	-95.00000*	7.57677	0	-110.5462	-79.4538
		10	-100.00000*	7.57677	0	-115.5462	-84.4538
		20	-100.00000*	7.57677	0	-115.5462	-84.4538
	0.1	0	5	7.57677	0.515	-10.5462	20.5462
		0.5	0	7.57677	1	-15.5462	15.5462
		1	0	7.57677	1	-15.5462	15.5462
		2	0	7.57677	1	-15.5462	15.5462
		4	-15	7.57677	0.058	-30.5462	0.5462
		8	-90.00000*	7.57677	0	-105.5462	-74.4538
		10	-95.00000*	7.57677	0	-110.5462	-79.4538
		20	-95.00000*	7.57677	0	-110.5462	-79.4538
	0.5	0	5	7.57677	0.515	-10.5462	20.5462
		0.1	0	7.57677	1	-15.5462	15.5462
		1	0	7.57677	1	-15.5462	15.5462
		2	0	7.57677	1	-15.5462	15.5462
		4	-15	7.57677	0.058	-30.5462	0.5462
		8	-90.00000*	7.57677	0	-105.5462	-74.4538
		10	-95.00000*	7.57677	0	-110.5462	-79.4538
		20	-95.00000*	7.57677	0	-110.5462	-79.4538
	1	0	5	7.57677	0.515	-10.5462	20.5462
		0.1	0	7.57677	1	-15.5462	15.5462
		0.5	0	7.57677	1	-15.5462	15.5462
		2	0	7.57677	1	-15.5462	15.5462
		4	-15	7.57677	0.058	-30.5462	0.5462
		8	-90.00000*	7.57677	0	-105.5462	-74.4538

	10	-95.00000*	7.57677	0	-110.5462	-79.4538
	20	-95.00000*	7.57677	0	-110.5462	-79.4538
2	0	5	7.57677	0.515	-10.5462	20.5462
	0.1	0	7.57677	1	-15.5462	15.5462
	0.5	0	7.57677	1	-15.5462	15.5462
	1	0	7.57677	1	-15.5462	15.5462
	4	-15	7.57677	0.058	-30.5462	0.5462
	8	-90.00000*	7.57677	0	-105.5462	-74.4538
	10	-95.00000*	7.57677	0	-110.5462	-79.4538
	20	-95.00000*	7.57677	0	-110.5462	-79.4538
4	0	20.00000*	7.57677	0.014	4.4538	35.5462
	0.1	15	7.57677	0.058	-0.5462	30.5462
	0.5	15	7.57677	0.058	-0.5462	30.5462
	1	15	7.57677	0.058	-0.5462	30.5462
	2	15	7.57677	0.058	-0.5462	30.5462
	8	-75.00000*	7.57677	0	-90.5462	-59.4538
	10	-80.00000*	7.57677	0	-95.5462	-64.4538
	20	-80.00000*	7.57677	0	-95.5462	-64.4538
8	0	95.00000*	7.57677	0	79.4538	110.5462
	0.1	90.00000*	7.57677	0	74.4538	105.5462
	0.5	90.00000*	7.57677	0	74.4538	105.5462
	1	90.00000*	7.57677	0	74.4538	105.5462
	2	90.00000*	7.57677	0	74.4538	105.5462
	4	75.00000*	7.57677	0	59.4538	90.5462
	10	-5	7.57677	0.515	-20.5462	10.5462
	20	-5	7.57677	0.515	-20.5462	10.5462
10	0	100.00000*	7.57677	0	84.4538	115.5462
	0.1	95.00000*	7.57677	0	79.4538	110.5462
	0.5	95.00000*	7.57677	0	79.4538	110.5462
	1	95.00000*	7.57677	0	79.4538	110.5462
	2	95.00000*	7.57677	0	79.4538	110.5462
	4	80.00000*	7.57677	0	64.4538	95.5462
	8	5	7.57677	0.515	-10.5462	20.5462
	20	0	7.57677	1	-15.5462	15.5462
20	0	100.00000*	7.57677	0	84.4538	115.5462
	0.1	95.00000*	7.57677	0	79.4538	110.5462
	0.5	95.00000*	7.57677	0	79.4538	110.5462

	1	95.00000*	7.57677	0	79.4538	110.5462
	2	95.00000*	7.57677	0	79.4538	110.5462
	4	80.00000*	7.57677	0	64.4538	95.5462
	8	5	7.57677	0.515	-10.5462	20.5462
	10	0	7.57677	1	-15.5462	15.5462

* The mean difference is significant at the 0.05 level.

Table S3. Post hoc tests results of 48-h acute toxicity test.

Multiple Comparisons							
Dependent Variable: Mortality							
	(I) Concentration	(J) Concentration	Mean Difference (I-J)	Std. Error	Sig.	95% Confidence Interval	
						Lower Bound	Upper Bound
LSD	0	0.1	-5	5.93171	0.407	-17.1709	7.1709
		0.5	-5	5.93171	0.407	-17.1709	7.1709
		1	-5	5.93171	0.407	-17.1709	7.1709
		2	-10	5.93171	0.103	-22.1709	2.1709
		4	-45.00000*	5.93171	0	-57.1709	-32.8291
		8	-95.00000*	5.93171	0	-107.1709	-82.8291
		10	-100.00000*	5.93171	0	-112.1709	-87.8291
		20	-100.00000*	5.93171	0	-112.1709	-87.8291
	0.1	0	5	5.93171	0.407	-7.1709	17.1709
		0.5	0	5.93171	1	-12.1709	12.1709
		1	0	5.93171	1	-12.1709	12.1709
		2	-5	5.93171	0.407	-17.1709	7.1709
		4	-40.00000*	5.93171	0	-52.1709	-27.8291
		8	-90.00000*	5.93171	0	-102.1709	-77.8291
		10	-95.00000*	5.93171	0	-107.1709	-82.8291
		20	-95.00000*	5.93171	0	-107.1709	-82.8291
	0.5	0	5	5.93171	0.407	-7.1709	17.1709
		0.1	0	5.93171	1	-12.1709	12.1709
		1	0	5.93171	1	-12.1709	12.1709
		2	-5	5.93171	0.407	-17.1709	7.1709
		4	-40.00000*	5.93171	0	-52.1709	-27.8291
		8	-90.00000*	5.93171	0	-102.1709	-77.8291
		10	-95.00000*	5.93171	0	-107.1709	-82.8291
		20	-95.00000*	5.93171	0	-107.1709	-82.8291
	1	0	5	5.93171	0.407	-7.1709	17.1709
		0.1	0	5.93171	1	-12.1709	12.1709
		0.5	0	5.93171	1	-12.1709	12.1709
		2	-5	5.93171	0.407	-17.1709	7.1709
		4	-40.00000*	5.93171	0	-52.1709	-27.8291
		8	-90.00000*	5.93171	0	-102.1709	-77.8291

	10	-95.00000*	5.93171	0	-107.1709	-82.8291
	20	-95.00000*	5.93171	0	-107.1709	-82.8291
2	0	10	5.93171	0.103	-2.1709	22.1709
	0.1	5	5.93171	0.407	-7.1709	17.1709
	0.5	5	5.93171	0.407	-7.1709	17.1709
	1	5	5.93171	0.407	-7.1709	17.1709
	4	-35.00000*	5.93171	0	-47.1709	-22.8291
	8	-85.00000*	5.93171	0	-97.1709	-72.8291
	10	-90.00000*	5.93171	0	-102.1709	-77.8291
	20	-90.00000*	5.93171	0	-102.1709	-77.8291
4	0	45.00000*	5.93171	0	32.8291	57.1709
	0.1	40.00000*	5.93171	0	27.8291	52.1709
	0.5	40.00000*	5.93171	0	27.8291	52.1709
	1	40.00000*	5.93171	0	27.8291	52.1709
	2	35.00000*	5.93171	0	22.8291	47.1709
	8	-50.00000*	5.93171	0	-62.1709	-37.8291
	10	-55.00000*	5.93171	0	-67.1709	-42.8291
	20	-55.00000*	5.93171	0	-67.1709	-42.8291
8	0	95.00000*	5.93171	0	82.8291	107.1709
	0.1	90.00000*	5.93171	0	77.8291	102.1709
	0.5	90.00000*	5.93171	0	77.8291	102.1709
	1	90.00000*	5.93171	0	77.8291	102.1709
	2	85.00000*	5.93171	0	72.8291	97.1709
	4	50.00000*	5.93171	0	37.8291	62.1709
	10	-5	5.93171	0.407	-17.1709	7.1709
	20	-5	5.93171	0.407	-17.1709	7.1709
10	0	100.00000*	5.93171	0	87.8291	112.1709
	0.1	95.00000*	5.93171	0	82.8291	107.1709
	0.5	95.00000*	5.93171	0	82.8291	107.1709
	1	95.00000*	5.93171	0	82.8291	107.1709
	2	90.00000*	5.93171	0	77.8291	102.1709
	4	55.00000*	5.93171	0	42.8291	67.1709
	8	5	5.93171	0.407	-7.1709	17.1709
	20	0	5.93171	1	-12.1709	12.1709
20	0	100.00000*	5.93171	0	87.8291	112.1709
	0.1	95.00000*	5.93171	0	82.8291	107.1709
	0.5	95.00000*	5.93171	0	82.8291	107.1709
	1	95.00000*	5.93171	0	82.8291	107.1709
	2	90.00000*	5.93171	0	77.8291	102.1709

	4	55.00000*	5.93171	0	42.8291	67.1709
	8	5	5.93171	0.40<br>7	-7.1709	17.1709
	10	0	5.93171	1	-12.1709	12.1709

* The mean difference is significant at the 0.05 level.

Table S4. Chi-square test result for acute toxic test.

			**Chi-Square**	**df**	**Sig.**
24-h	LOGIT	Pearson Goodness-of-Fit Test	319.998	38	0
48-h	LOGIT	Pearson Goodness-of-Fit Test	133.112	38	0

Table S5. Confidence limits of probit analysis (24 h).

Confidence Limits							
	Probability	95% Confidence Limits for CON			95% Confidence Limits for log(CON)b		
		Estimate	Lower Bound	Upper Bound	Estimate	Lower Bound	Upper Bound
LOGITa	0.01	0.615	.	.	-0.211	.	.
	0.02	0.824	.	.	-0.084	.	.
	0.03	0.979	.	.	-0.009	.	.
	0.04	1.109	.	.	0.045	.	.
	0.05	1.222	.	.	0.087	.	.
	0.06	1.325	.	.	0.122	.	.
	0.07	1.419	.	.	0.152	.	.
	0.08	1.507	.	.	0.178	.	.
	0.09	1.59	.	.	0.201	.	.
	0.1	1.669	.	.	0.222	.	.
	0.15	2.023	.	.	0.306	.	.
	0.2	2.339	.	.	0.369	.	.
	0.25	2.637	.	.	0.421	.	.
	0.3	2.928	.	.	0.467	.	.
	0.35	3.22	.	.	0.508	.	.
	0.4	3.52	.	.	0.546	.	.
	0.45	3.833	.	.	0.584	.	.
	0.5	4.167	.	.	0.62	.	.
	0.55	4.531	.	.	0.656	.	.
	0.6	4.934	.	.	0.693	.	.
	0.65	5.393	.	.	0.732	.	.
	0.7	5.931	.	.	0.773	.	.
	0.75	6.586	.	.	0.819	.	.
	0.8	7.424	.	.	0.871	.	.
	0.85	8.583	.	.	0.934	.	.
	0.9	10.408	.	.	1.017	.	.
	0.91	10.925	.	.	1.038	.	.
	0.92	11.527	.	.	1.062	.	.
	0.93	12.241	.	.	1.088	.	.
	0.94	13.111	.	.	1.118	.	.
	0.95	14.208	.	.	1.153	.	.
	0.96	15.66	.	.	1.195	.	.
	0.97	17.73	.	.	1.249	.	.
	0.98	21.083	.	.	1.324	.	.
	0.99	28.26	.	.	1.451	.	.

a A heterogeneity factor is used.
b Logarithm base = 10.

Table SS6 Confidence limits of probit analysis (48 h).

Confidence Limits							
	Probability	95% Confidence Limits for CON			95% Confidence Limits for log(CON)b		
		Estimate	Lower Bound	Upper Bound	Estimate	Lower Bound	Upper Bound
LOGITa	0.01	0.459	.	.	-0.338	.	.
	0.02	0.624	.	.	-0.205	.	.
	0.03	0.749	.	.	-0.125	.	.
	0.04	0.854	.	.	-0.069	.	.
	0.05	0.946	.	.	-0.024	.	.
	0.06	1.029	.	.	0.012	.	.
	0.07	1.106	.	.	0.044	.	.
	0.08	1.179	.	.	0.071	.	.
	0.09	1.247	.	.	0.096	.	.
	0.1	1.312	.	.	0.118	.	.
	0.15	1.607	.	.	0.206	.	.
	0.2	1.872	.	.	0.272	.	.
	0.25	2.124	.	.	0.327	.	.
	0.3	2.371	.	.	0.375	.	.
	0.35	2.621	.	.	0.418	.	.
	0.4	2.878	.	.	0.459	.	.
	0.45	3.148	.	.	0.498	.	.
	0.5	3.438	.	.	0.536	.	.
	0.55	3.754	.	.	0.574	.	.
	0.6	4.106	.	.	0.613	.	.
	0.65	4.509	.	.	0.654	.	.
	0.7	4.983	.	.	0.698	.	.
	0.75	5.564	.	.	0.745	.	.
	0.8	6.311	.	.	0.8	.	.
	0.85	7.352	.	.	0.866	.	.
	0.9	9.005	.	.	0.954	.	.
	0.91	9.477	.	.	0.977	.	.
	0.92	10.027	.	.	1.001	.	.
	0.93	10.681	.	.	1.029	.	.
	0.94	11.482	.	.	1.06	.	.
	0.95	12.495	.	.	1.097	.	.
	0.96	13.842	.	.	1.141	.	.
	0.97	15.774	.	.	1.198	.	.
	0.98	18.926	.	.	1.277	.	.
	0.99	25.76	.	.	1.411	.	.

a A heterogeneity factor is used.

b Logarithm base = 10.

Table S7. ANOVA results of fourth-instar larvae mortality rate.

		Sum of Squares	df	Mean Square	F	Sig.
24 h	Between Groups	0	5	0	.	.
	Within Groups	0	12	0		
	Total	0	17			
48 h	Between Groups	3644.444	5	728.889	6.56	0.004
	Within Groups	1333.333	12	111.111		
	Total	4977.778	17			
72 h	Between Groups	7844.444	5	1568.889	23.533	0
	Within Groups	800	12	66.667		
	Total	8644.444	17			
96 h	Between Groups	7844.444	5	1568.889	23.533	0
	Within Groups	800	12	66.667		
	Total	8644.444	17			
120 h	Between Groups	7844.444	5	1568.889	23.533	0
	Within Groups	800	12	66.667		
	Total	8644.444	17			
136 h	Between Groups	7844.444	5	1568.889	35.3	0
	Within Groups	533.333	12	44.444		
	Total	8377.778	17			
144 h	Between Groups	6844.444	5	1368.889	15.4	0
	Within Groups	1066.667	12	88.889		
	Total	7911.111	17			
160 h	Between Groups	7133.333	5	1426.667	16.05	0
	Within Groups	1066.667	12	88.889		
	Total	8200	17			
168 h	Between Groups	7377.778	5	1475.556	16.6	0
	Within Groups	1066.667	12	88.889		
	Total	8444.444	17			
192 h	Between Groups	7466.667	5	1493.333	8.4	0.001
	Within Groups	2133.333	12	177.778		
	Total	9600	17			
216 h	Between Groups	7377.778	5	1475.556	7.378	0.002
	Within Groups	2400	12	200		
	Total	9777.778	17			
240 h	Between Groups	8177.778	5	1635.556	6.133	0.005
	Within Groups	3200	12	266.667		
	Total	11377.778	17			

Table S8. Post hoc tests results of fourth-instar larvae mortality rate.

Multiple Comparisons								
Dependent Variable		(I) Concentration	(J) Concentration	Mean Difference (I-J)	Std. Error	Sig.	95% Confidence Interval	
							Lower Bound	Upper Bound
Mortality48	LSD	0	1.5	0	8.60663	1	-18.7522	18.7522
			2	0	8.60663	1	-18.7522	18.7522
			2.5	-6.66667	8.60663	0.454	-25.4189	12.0856
			3	-6.66667	8.60663	0.454	-25.4189	12.0856
			4	-40.00000*	8.60663	0.001	-58.7522	-21.2478
		1.5	0	0	8.60663	1	-18.7522	18.7522
			2	0	8.60663	1	-18.7522	18.7522
			2.5	-6.66667	8.60663	0.454	-25.4189	12.0856
			3	-6.66667	8.60663	0.454	-25.4189	12.0856
			4	-40.00000*	8.60663	0.001	-58.7522	-21.2478
		2	0	0	8.60663	1	-18.7522	18.7522
			1.5	0	8.60663	1	-18.7522	18.7522
			2.5	-6.66667	8.60663	0.454	-25.4189	12.0856
			3	-6.66667	8.60663	0.454	-25.4189	12.0856
			4	-40.00000*	8.60663	0.001	-58.7522	-21.2478
		2.5	0	6.66667	8.60663	0.454	-12.0856	25.4189
			1.5	6.66667	8.60663	0.454	-12.0856	25.4189
			2	6.66667	8.60663	0.454	-12.0856	25.4189
			3	0	8.60663	1	-18.7522	18.7522
			4	-33.33333*	8.60663	0.002	-52.0856	-14.5811
		3	0	6.66667	8.60663	0.454	-12.0856	25.4189
			1.5	6.66667	8.60663	0.454	-12.0856	25.4189
			2	6.66667	8.60663	0.454	-12.0856	25.4189
			2.5	0	8.60663	1	-18.7522	18.7522
			4	-33.33333*	8.60663	0.002	-52.0856	-14.5811
		4	0	40.00000*	8.60663	0.001	21.2478	58.7522
			1.5	40.00000*	8.60663	0.001	21.2478	58.7522
			2	40.00000*	8.60663	0.001	21.2478	58.7522
			2.5	33.33333*	8.60663	0.002	14.5811	52.0856
			3	33.33333*	8.60663	0.002	14.5811	52.0856
Mortality72	LSD	0	1.5	0	6.66667	1	-14.5254	14.5254
			2	-6.66667	6.66667	0.337	-21.1921	7.8588
			2.5	-6.66667	6.66667	0.337	-21.1921	7.8588
			3	-13.33333	6.66667	0.069	-27.8588	1.1921
			4	-60.00000*	6.66667	0	-74.5254	-45.4746
		1.5	0	0	6.66667	1	-14.5254	14.5254
			2	-6.66667	6.66667	0.337	-21.1921	7.8588
			2.5	-6.66667	6.66667	0.337	-21.1921	7.8588
			3	-13.33333	6.66667	0.069	-27.8588	1.1921
			4	-60.00000*	6.66667	0	-74.5254	-45.4746
		2	0	6.66667	6.66667	0.337	-7.8588	21.1921
			1.5	6.66667	6.66667	0.337	-7.8588	21.1921
			2.5	0	6.66667	1	-14.5254	14.5254
			3	-6.66667	6.66667	0.337	-21.1921	7.8588
			4	-53.33333*	6.66667	0	-67.8588	-38.8079
		2.5	0	6.66667	6.66667	0.337	-7.8588	21.1921

			1.5	6.66667	6.66667	0.337	-7.8588	21.1921
			2	0	6.66667	1	-14.5254	14.5254
			3	-6.66667	6.66667	0.337	-21.1921	7.8588
			4	-53.33333*	6.66667	0	-67.8588	-38.8079
		3	0	13.33333	6.66667	0.069	-1.1921	27.8588
			1.5	13.33333	6.66667	0.069	-1.1921	27.8588
			2	6.66667	6.66667	0.337	-7.8588	21.1921
			2.5	6.66667	6.66667	0.337	-7.8588	21.1921
			4	-46.66667*	6.66667	0	-61.1921	-32.1412
		4	0	60.00000*	6.66667	0	45.4746	74.5254
			1.5	60.00000*	6.66667	0	45.4746	74.5254
			2	53.33333*	6.66667	0	38.8079	67.8588
			2.5	53.33333*	6.66667	0	38.8079	67.8588
			3	46.66667*	6.66667	0	32.1412	61.1921
Mortality96	LSD	0	1.5	0	6.66667	1	-14.5254	14.5254
			2	-6.66667	6.66667	0.337	-21.1921	7.8588
			2.5	-6.66667	6.66667	0.337	-21.1921	7.8588
			3	-13.33333	6.66667	0.069	-27.8588	1.1921
			4	-60.00000*	6.66667	0	-74.5254	-45.4746
		1.5	0	0	6.66667	1	-14.5254	14.5254
			2	-6.66667	6.66667	0.337	-21.1921	7.8588
			2.5	-6.66667	6.66667	0.337	-21.1921	7.8588
			3	-13.33333	6.66667	0.069	-27.8588	1.1921
			4	-60.00000*	6.66667	0	-74.5254	-45.4746
		2	0	6.66667	6.66667	0.337	-7.8588	21.1921
			1.5	6.66667	6.66667	0.337	-7.8588	21.1921
			2.5	0	6.66667	1	-14.5254	14.5254
			3	-6.66667	6.66667	0.337	-21.1921	7.8588
			4	-53.33333*	6.66667	0	-67.8588	-38.8079
		2.5	0	6.66667	6.66667	0.337	-7.8588	21.1921
			1.5	6.66667	6.66667	0.337	-7.8588	21.1921
			2	0	6.66667	1	-14.5254	14.5254
			3	-6.66667	6.66667	0.337	-21.1921	7.8588
			4	-53.33333*	6.66667	0	-67.8588	-38.8079
		3	0	13.33333	6.66667	0.069	-1.1921	27.8588
			1.5	13.33333	6.66667	0.069	-1.1921	27.8588
			2	6.66667	6.66667	0.337	-7.8588	21.1921
			2.5	6.66667	6.66667	0.337	-7.8588	21.1921
			4	-46.66667*	6.66667	0	-61.1921	-32.1412
		4	0	60.00000*	6.66667	0	45.4746	74.5254
			1.5	60.00000*	6.66667	0	45.4746	74.5254
			2	53.33333*	6.66667	0	38.8079	67.8588
			2.5	53.33333*	6.66667	0	38.8079	67.8588
			3	46.66667*	6.66667	0	32.1412	61.1921
Mortality120	LSD	0	1.5	0	6.66667	1	-14.5254	14.5254
			2	-6.66667	6.66667	0.337	-21.1921	7.8588
			2.5	-6.66667	6.66667	0.337	-21.1921	7.8588
			3	-13.33333	6.66667	0.069	-27.8588	1.1921
			4	-60.00000*	6.66667	0	-74.5254	-45.4746
		1.5	0	0	6.66667	1	-14.5254	14.5254
			2	-6.66667	6.66667	0.337	-21.1921	7.8588
			2.5	-6.66667	6.66667	0.337	-21.1921	7.8588

			3	-13.33333	6.66667	0.069	-27.8588	1.1921
			4	-60.00000*	6.66667	0	-74.5254	-45.4746
		2	0	6.66667	6.66667	0.337	-7.8588	21.1921
			1.5	6.66667	6.66667	0.337	-7.8588	21.1921
			2.5	0	6.66667	1	-14.5254	14.5254
			3	-6.66667	6.66667	0.337	-21.1921	7.8588
			4	-53.33333*	6.66667	0	-67.8588	-38.8079
		2.5	0	6.66667	6.66667	0.337	-7.8588	21.1921
			1.5	6.66667	6.66667	0.337	-7.8588	21.1921
			2	0	6.66667	1	-14.5254	14.5254
			3	-6.66667	6.66667	0.337	-21.1921	7.8588
			4	-53.33333*	6.66667	0	-67.8588	-38.8079
		3	0	13.33333	6.66667	0.069	-1.1921	27.8588
			1.5	13.33333	6.66667	0.069	-1.1921	27.8588
			2	6.66667	6.66667	0.337	-7.8588	21.1921
			2.5	6.66667	6.66667	0.337	-7.8588	21.1921
			4	-46.66667*	6.66667	0	-61.1921	-32.1412
		4	0	60.00000*	6.66667	0	45.4746	74.5254
			1.5	60.00000*	6.66667	0	45.4746	74.5254
			2	53.33333*	6.66667	0	38.8079	67.8588
			2.5	53.33333*	6.66667	0	38.8079	67.8588
			3	46.66667*	6.66667	0	32.1412	61.1921
Mortality136	LSD	0	1.5	0	5.44331	1	-11.86	11.86
			2	-6.66667	5.44331	0.244	-18.5266	5.1933
			2.5	-20.00000*	5.44331	0.003	-31.86	-8.14
			3	-26.66667*	5.44331	0	-38.5266	-14.8067
			4	-60.00000*	5.44331	0	-71.86	-48.14
		1.5	0	0	5.44331	1	-11.86	11.86
			2	-6.66667	5.44331	0.244	-18.5266	5.1933
			2.5	-20.00000*	5.44331	0.003	-31.86	-8.14
			3	-26.66667*	5.44331	0	-38.5266	-14.8067
			4	-60.00000*	5.44331	0	-71.86	-48.14
		2	0	6.66667	5.44331	0.244	-5.1933	18.5266
			1.5	6.66667	5.44331	0.244	-5.1933	18.5266
			2.5	-13.33333*	5.44331	0.031	-25.1933	-1.4734
			3	-20.00000*	5.44331	0.003	-31.86	-8.14
			4	-53.33333*	5.44331	0	-65.1933	-41.4734
		2.5	0	20.00000*	5.44331	0.003	8.14	31.86
			1.5	20.00000*	5.44331	0.003	8.14	31.86
			2	13.33333*	5.44331	0.031	1.4734	25.1933
			3	-6.66667	5.44331	0.244	-18.5266	5.1933
			4	-40.00000*	5.44331	0	-51.86	-28.14
		3	0	26.66667*	5.44331	0	14.8067	38.5266
			1.5	26.66667*	5.44331	0	14.8067	38.5266
			2	20.00000*	5.44331	0.003	8.14	31.86
			2.5	6.66667	5.44331	0.244	-5.1933	18.5266
			4	-33.33333*	5.44331	0	-45.1933	-21.4734
		4	0	60.00000*	5.44331	0	48.14	71.86
			1.5	60.00000*	5.44331	0	48.14	71.86
			2	53.33333*	5.44331	0	41.4734	65.1933
			2.5	40.00000*	5.44331	0	28.14	51.86
			3	33.33333*	5.44331	0	21.4734	45.1933

Mortality144	LSD	0	1.5	-6.66667	7.698	0.403	-23.4392	10.1058
			2	-13.33333	7.698	0.109	-30.1058	3.4392
			2.5	-26.66667*	7.698	0.005	-43.4392	-9.8942
			3	-26.66667*	7.698	0.005	-43.4392	-9.8942
			4	-60.00000*	7.698	0	-76.7725	-43.2275
		1.5	0	6.66667	7.698	0.403	-10.1058	23.4392
			2	-6.66667	7.698	0.403	-23.4392	10.1058
			2.5	-20.00000*	7.698	0.023	-36.7725	-3.2275
			3	-20.00000*	7.698	0.023	-36.7725	-3.2275
			4	-53.33333*	7.698	0	-70.1058	-36.5608
		2	0	13.33333	7.698	0.109	-3.4392	30.1058
			1.5	6.66667	7.698	0.403	-10.1058	23.4392
			2.5	-13.33333	7.698	0.109	-30.1058	3.4392
			3	-13.33333	7.698	0.109	-30.1058	3.4392
			4	-46.66667*	7.698	0	-63.4392	-29.8942
		2.5	0	26.66667*	7.698	0.005	9.8942	43.4392
			1.5	20.00000*	7.698	0.023	3.2275	36.7725
			2	13.33333	7.698	0.109	-3.4392	30.1058
			3	0	7.698	1	-16.7725	16.7725
			4	-33.33333*	7.698	0.001	-50.1058	-16.5608
		3	0	26.66667*	7.698	0.005	9.8942	43.4392
			1.5	20.00000*	7.698	0.023	3.2275	36.7725
			2	13.33333	7.698	0.109	-3.4392	30.1058
			2.5	0	7.698	1	-16.7725	16.7725
			4	-33.33333*	7.698	0.001	-50.1058	-16.5608
		4	0	60.00000*	7.698	0	43.2275	76.7725
			1.5	53.33333*	7.698	0	36.5608	70.1058
			2	46.66667*	7.698	0	29.8942	63.4392
			2.5	33.33333*	7.698	0.001	16.5608	50.1058
			3	33.33333*	7.698	0.001	16.5608	50.1058
Mortality160	LSD	0	1.5	-6.66667	7.698	0.403	-23.4392	10.1058
			2	-13.33333	7.698	0.109	-30.1058	3.4392
			2.5	-33.33333*	7.698	0.001	-50.1058	-16.5608
			3	-26.66667*	7.698	0.005	-43.4392	-9.8942
			4	-60.00000*	7.698	0	-76.7725	-43.2275
		1.5	0	6.66667	7.698	0.403	-10.1058	23.4392
			2	-6.66667	7.698	0.403	-23.4392	10.1058
			2.5	-26.66667*	7.698	0.005	-43.4392	-9.8942
			3	-20.00000*	7.698	0.023	-36.7725	-3.2275
			4	-53.33333*	7.698	0	-70.1058	-36.5608
		2	0	13.33333	7.698	0.109	-3.4392	30.1058
			1.5	6.66667	7.698	0.403	-10.1058	23.4392
			2.5	-20.00000*	7.698	0.023	-36.7725	-3.2275
			3	-13.33333	7.698	0.109	-30.1058	3.4392
			4	-46.66667*	7.698	0	-63.4392	-29.8942
		2.5	0	33.33333*	7.698	0.001	16.5608	50.1058
			1.5	26.66667*	7.698	0.005	9.8942	43.4392
			2	20.00000*	7.698	0.023	3.2275	36.7725
			3	6.66667	7.698	0.403	-10.1058	23.4392
			4	-26.66667*	7.698	0.005	-43.4392	-9.8942
		3	0	26.66667*	7.698	0.005	9.8942	43.4392
			1.5	20.00000*	7.698	0.023	3.2275	36.7725

			2	13.33333	7.698	0.109	-3.4392	30.1058
			2.5	-6.66667	7.698	0.403	-23.4392	10.1058
			4	-33.33333*	7.698	0.001	-50.1058	-16.5608
		4	0	60.00000*	7.698	0	43.2275	76.7725
			1.5	53.33333*	7.698	0	36.5608	70.1058
			2	46.66667*	7.698	0	29.8942	63.4392
			2.5	26.66667*	7.698	0.005	9.8942	43.4392
			3	33.33333*	7.698	0.001	16.5608	50.1058
Mortality168	LSD	0	1.5	-6.66667	7.698	0.403	-23.4392	10.1058
			2	-13.33333	7.698	0.109	-30.1058	3.4392
			2.5	-33.33333*	7.698	0.001	-50.1058	-16.5608
			3	-33.33333*	7.698	0.001	-50.1058	-16.5608
			4	-60.00000*	7.698	0	-76.7725	-43.2275
		1.5	0	6.66667	7.698	0.403	-10.1058	23.4392
			2	-6.66667	7.698	0.403	-23.4392	10.1058
			2.5	-26.66667*	7.698	0.005	-43.4392	-9.8942
			3	-26.66667*	7.698	0.005	-43.4392	-9.8942
			4	-53.33333*	7.698	0	-70.1058	-36.5608
		2	0	13.33333	7.698	0.109	-3.4392	30.1058
			1.5	6.66667	7.698	0.403	-10.1058	23.4392
			2.5	-20.00000*	7.698	0.023	-36.7725	-3.2275
			3	-20.00000*	7.698	0.023	-36.7725	-3.2275
			4	-46.66667*	7.698	0	-63.4392	-29.8942
		2.5	0	33.33333*	7.698	0.001	16.5608	50.1058
			1.5	26.66667*	7.698	0.005	9.8942	43.4392
			2	20.00000*	7.698	0.023	3.2275	36.7725
			3	0	7.698	1	-16.7725	16.7725
			4	-26.66667*	7.698	0.005	-43.4392	-9.8942
		3	0	33.33333*	7.698	0.001	16.5608	50.1058
			1.5	26.66667*	7.698	0.005	9.8942	43.4392
			2	20.00000*	7.698	0.023	3.2275	36.7725
			2.5	0	7.698	1	-16.7725	16.7725
			4	-26.66667*	7.698	0.005	-43.4392	-9.8942
		4	0	60.00000*	7.698	0	43.2275	76.7725
			1.5	53.33333*	7.698	0	36.5608	70.1058
			2	46.66667*	7.698	0	29.8942	63.4392
			2.5	26.66667*	7.698	0.005	9.8942	43.4392
			3	26.66667*	7.698	0.005	9.8942	43.4392
Mortality192	LSD	0	1.5	-6.66667	10.88662	0.552	-30.3866	17.0532
			2	-20	10.88662	0.091	-43.7199	3.7199
			2.5	-33.33333*	10.88662	0.01	-57.0532	-9.6134
			3	-40.00000*	10.88662	0.003	-63.7199	-16.2801
			4	-60.00000*	10.88662	0	-83.7199	-36.2801
		1.5	0	6.66667	10.88662	0.552	-17.0532	30.3866
			2	-13.33333	10.88662	0.244	-37.0532	10.3866
			2.5	-26.66667*	10.88662	0.031	-50.3866	-2.9468
			3	-33.33333*	10.88662	0.01	-57.0532	-9.6134
			4	-53.33333*	10.88662	0	-77.0532	-29.6134
		2	0	20	10.88662	0.091	-3.7199	43.7199
			1.5	13.33333	10.88662	0.244	-10.3866	37.0532
			2.5	-13.33333	10.88662	0.244	-37.0532	10.3866
			3	-20	10.88662	0.091	-43.7199	3.7199

			4	-40.00000*	10.88662	0.003	-63.7199	-16.2801
		2.5	0	33.33333*	10.88662	0.01	9.6134	57.0532
			1.5	26.66667*	10.88662	0.031	2.9468	50.3866
			2	13.33333	10.88662	0.244	-10.3866	37.0532
			3	-6.66667	10.88662	0.552	-30.3866	17.0532
			4	-26.66667*	10.88662	0.031	-50.3866	-2.9468
		3	0	40.00000*	10.88662	0.003	16.2801	63.7199
			1.5	33.33333*	10.88662	0.01	9.6134	57.0532
			2	20	10.88662	0.091	-3.7199	43.7199
			2.5	6.66667	10.88662	0.552	-17.0532	30.3866
			4	-20	10.88662	0.091	-43.7199	3.7199
		4	0	60.00000*	10.88662	0	36.2801	83.7199
			1.5	53.33333*	10.88662	0	29.6134	77.0532
			2	40.00000*	10.88662	0.003	16.2801	63.7199
			2.5	26.66667*	10.88662	0.031	2.9468	50.3866
			3	20	10.88662	0.091	-3.7199	43.7199
Mortality216	LSD	0	1.5	-6.66667	11.54701	0.574	-31.8254	18.4921
			2	-33.33333*	11.54701	0.014	-58.4921	-8.1746
			2.5	-33.33333*	11.54701	0.014	-58.4921	-8.1746
			3	-40.00000*	11.54701	0.005	-65.1588	-14.8412
			4	-60.00000*	11.54701	0	-85.1588	-34.8412
		1.5	0	6.66667	11.54701	0.574	-18.4921	31.8254
			2	-26.66667*	11.54701	0.04	-51.8254	-1.5079
			2.5	-26.66667*	11.54701	0.04	-51.8254	-1.5079
			3	-33.33333*	11.54701	0.014	-58.4921	-8.1746
			4	-53.33333*	11.54701	0.001	-78.4921	-28.1746
		2	0	33.33333*	11.54701	0.014	8.1746	58.4921
			1.5	26.66667*	11.54701	0.04	1.5079	51.8254
			2.5	0	11.54701	1	-25.1588	25.1588
			3	-6.66667	11.54701	0.574	-31.8254	18.4921
			4	-26.66667*	11.54701	0.04	-51.8254	-1.5079
		2.5	0	33.33333*	11.54701	0.014	8.1746	58.4921
			1.5	26.66667*	11.54701	0.04	1.5079	51.8254
			2	0	11.54701	1	-25.1588	25.1588
			3	-6.66667	11.54701	0.574	-31.8254	18.4921
			4	-26.66667*	11.54701	0.04	-51.8254	-1.5079
		3	0	40.00000*	11.54701	0.005	14.8412	65.1588
			1.5	33.33333*	11.54701	0.014	8.1746	58.4921
			2	6.66667	11.54701	0.574	-18.4921	31.8254
			2.5	6.66667	11.54701	0.574	-18.4921	31.8254
			4	-20	11.54701	0.109	-45.1588	5.1588
		4	0	60.00000*	11.54701	0	34.8412	85.1588
			1.5	53.33333*	11.54701	0.001	28.1746	78.4921
			2	26.66667*	11.54701	0.04	1.5079	51.8254
			2.5	26.66667*	11.54701	0.04	1.5079	51.8254
			3	20	11.54701	0.109	-5.1588	45.1588
Mortality240	LSD	0	1.5	-6.66667	13.33333	0.626	-35.7175	22.3842
			2	-33.33333*	13.33333	0.028	-62.3842	-4.2825
			2.5	-40.00000*	13.33333	0.011	-69.0508	-10.9492
			3	-46.66667*	13.33333	0.004	-75.7175	-17.6158
			4	-60.00000*	13.33333	0.001	-89.0508	-30.9492
		1.5	0	6.66667	13.33333	0.626	-22.3842	35.7175

	2	-26.66667	13.33333	0.069	-55.7175	2.3842
	2.5	-33.33333*	13.33333	0.028	-62.3842	-4.2825
	3	-40.00000*	13.33333	0.011	-69.0508	-10.9492
	4	-53.33333*	13.33333	0.002	-82.3842	-24.2825
2	0	33.33333*	13.33333	0.028	4.2825	62.3842
	1.5	26.66667	13.33333	0.069	-2.3842	55.7175
	2.5	-6.66667	13.33333	0.626	-35.7175	22.3842
	3	-13.33333	13.33333	0.337	-42.3842	15.7175
	4	-26.66667	13.33333	0.069	-55.7175	2.3842
2.5	0	40.00000*	13.33333	0.011	10.9492	69.0508
	1.5	33.33333*	13.33333	0.028	4.2825	62.3842
	2	6.66667	13.33333	0.626	-22.3842	35.7175
	3	-6.66667	13.33333	0.626	-35.7175	22.3842
	4	-20	13.33333	0.159	-49.0508	9.0508
3	0	46.66667*	13.33333	0.004	17.6158	75.7175
	1.5	40.00000*	13.33333	0.011	10.9492	69.0508
	2	13.33333	13.33333	0.337	-15.7175	42.3842
	2.5	6.66667	13.33333	0.626	-22.3842	35.7175
	4	-13.33333	13.33333	0.337	-42.3842	15.7175
4	0	60.00000*	13.33333	0.001	30.9492	89.0508
	1.5	53.33333*	13.33333	0.002	24.2825	82.3842
	2	26.66667	13.33333	0.069	-2.3842	55.7175
	2.5	20	13.33333	0.159	-9.0508	49.0508
	3	13.33333	13.33333	0.337	-15.7175	42.3842

* The mean difference is significant at the 0.05 level.

Table S9. ANOVA results of cumulative pupation rate.

		Sum of Squares	df	Mean Square	F	Sig.
24 h	Between Groups	0	5	0	.	.
	Within Groups	0	12	0		
	Total	0	17			
48 h	Between Groups	0	5	0	.	.
	Within Groups	0	12	0		
	Total	0	17			
72 h	Between Groups	0	5	0	.	.
	Within Groups	0	12	0		
	Total	0	17			
96 h	Between Groups	0	5	0	.	.
	Within Groups	0	12	0		
	Total	0	17			
120 h	Between Groups	111.111	5	22.222	1	0.458
	Within Groups	266.667	12	22.222		
	Total	377.778	17			
136 h	Between Groups	2311.111	5	462.222	2.971	0.057
	Within Groups	1866.667	12	155.556		
	Total	4177.778	17			
144 h	Between Groups	2577.778	5	515.556	2.9	0.061
	Within Groups	2133.333	12	177.778		
	Total	4711.111	17			
160 h	Between Groups	8977.778	5	1795.556	8.08	0.002
	Within Groups	2666.667	12	222.222		
	Total	11644.444	17			
168 h	Between Groups	8733.333	5	1746.667	6.046	0.005
	Within Groups	3466.667	12	288.889		
	Total	12200	17			
192 h	Between Groups	9600	5	1920	7.2	0.002
	Within Groups	3200	12	266.667		
	Total	12800	17			
216 h	Between Groups	10244.444	5	2048.889	6.147	0.005
	Within Groups	4000	12	333.333		
	Total	14244.444	17			
240 h	Between Groups	8177.778	5	1635.556	6.133	0.005
	Within Groups	3200	12	266.667		
	Total	11377.778	17			

Table S10. Post hoc tests results of cumulative pupation rate.

**Multiple Comparisons**								
**Dependent Variable**		(I) Concentration	(J) Concentration	Mean Difference (I-J)	Std. Error	Sig.	95% Confidence Interval	
							Lower Bound	Upper Bound
Pupation120	LSD	0	1.5	6.66667	3.849	0.109	-1.7196	15.0529
			2	6.66667	3.849	0.109	-1.7196	15.0529
			2.5	6.66667	3.849	0.109	-1.7196	15.0529
			3	6.66667	3.849	0.109	-1.7196	15.0529
			4	6.66667	3.849	0.109	-1.7196	15.0529
		1.5	0	-6.66667	3.849	0.109	-15.0529	1.7196
			2	0	3.849	1	-8.3863	8.3863
			2.5	0	3.849	1	-8.3863	8.3863
			3	0	3.849	1	-8.3863	8.3863
			4	0	3.849	1	-8.3863	8.3863
		2	0	-6.66667	3.849	0.109	-15.0529	1.7196
			1.5	0	3.849	1	-8.3863	8.3863
			2.5	0	3.849	1	-8.3863	8.3863
			3	0	3.849	1	-8.3863	8.3863
			4	0	3.849	1	-8.3863	8.3863
		2.5	0	-6.66667	3.849	0.109	-15.0529	1.7196
			1.5	0	3.849	1	-8.3863	8.3863
			2	0	3.849	1	-8.3863	8.3863
			3	0	3.849	1	-8.3863	8.3863
			4	0	3.849	1	-8.3863	8.3863
		3	0	-6.66667	3.849	0.109	-15.0529	1.7196
			1.5	0	3.849	1	-8.3863	8.3863
			2	0	3.849	1	-8.3863	8.3863
			2.5	0	3.849	1	-8.3863	8.3863
			4	0	3.849	1	-8.3863	8.3863
		4	0	-6.66667	3.849	0.109	-15.0529	1.7196
			1.5	0	3.849	1	-8.3863	8.3863
			2	0	3.849	1	-8.3863	8.3863
			2.5	0	3.849	1	-8.3863	8.3863
			3	0	3.849	1	-8.3863	8.3863
Pupation136	LSD	0	1.5	20	10.1835	0.073	-2.1879	42.1879
			2	20	10.1835	0.073	-2.1879	42.1879
			2.5	33.33333*	10.1835	0.007	11.1454	55.5213
			3	33.33333*	10.1835	0.007	11.1454	55.5213
			4	26.66667*	10.1835	0.022	4.4787	48.8546
		1.5	0	-20	10.1835	0.073	-42.1879	2.1879
			2	0	10.1835	1	-22.1879	22.1879
			2.5	13.33333	10.1835	0.215	-8.8546	35.5213
			3	13.33333	10.1835	0.215	-8.8546	35.5213
			4	6.66667	10.1835	0.525	-15.5213	28.8546
		2	0	-20	10.1835	0.073	-42.1879	2.1879
			1.5	0	10.1835	1	-22.1879	22.1879
			2.5	13.33333	10.1835	0.215	-8.8546	35.5213
			3	13.33333	10.1835	0.215	-8.8546	35.5213
			4	6.66667	10.1835	0.525	-15.5213	28.8546
		2.5	0	-33.33333*	10.1835	0.007	-55.5213	-11.1454

			1.5	-13.33333	10.1835	0.215	-35.5213	8.8546
			2	-13.33333	10.1835	0.215	-35.5213	8.8546
			3	0	10.1835	1	-22.1879	22.1879
			4	-6.66667	10.1835	0.525	-28.8546	15.5213
		3	0	-33.33333*	10.1835	0.007	-55.5213	-11.1454
			1.5	-13.33333	10.1835	0.215	-35.5213	8.8546
			2	-13.33333	10.1835	0.215	-35.5213	8.8546
			2.5	0	10.1835	1	-22.1879	22.1879
			4	-6.66667	10.1835	0.525	-28.8546	15.5213
		4	0	-26.66667*	10.1835	0.022	-48.8546	-4.4787
			1.5	-6.66667	10.1835	0.525	-28.8546	15.5213
			2	-6.66667	10.1835	0.525	-28.8546	15.5213
			2.5	6.66667	10.1835	0.525	-15.5213	28.8546
			3	6.66667	10.1835	0.525	-15.5213	28.8546
Pupation144	LSD	0	1.5	13.33333	10.88662	0.244	-10.3866	37.0532
			2	26.66667*	10.88662	0.031	2.9468	50.3866
			2.5	33.33333*	10.88662	0.01	9.6134	57.0532
			3	33.33333*	10.88662	0.01	9.6134	57.0532
			4	26.66667*	10.88662	0.031	2.9468	50.3866
		1.5	0	-13.33333	10.88662	0.244	-37.0532	10.3866
			2	13.33333	10.88662	0.244	-10.3866	37.0532
			2.5	20	10.88662	0.091	-3.7199	43.7199
			3	20	10.88662	0.091	-3.7199	43.7199
			4	13.33333	10.88662	0.244	-10.3866	37.0532
		2	0	-26.66667*	10.88662	0.031	-50.3866	-2.9468
			1.5	-13.33333	10.88662	0.244	-37.0532	10.3866
			2.5	6.66667	10.88662	0.552	-17.0532	30.3866
			3	6.66667	10.88662	0.552	-17.0532	30.3866
			4	0	10.88662	1	-23.7199	23.7199
		2.5	0	-33.33333*	10.88662	0.01	-57.0532	-9.6134
			1.5	-20	10.88662	0.091	-43.7199	3.7199
			2	-6.66667	10.88662	0.552	-30.3866	17.0532
			3	0	10.88662	1	-23.7199	23.7199
			4	-6.66667	10.88662	0.552	-30.3866	17.0532
		3	0	-33.33333*	10.88662	0.01	-57.0532	-9.6134
			1.5	-20	10.88662	0.091	-43.7199	3.7199
			2	-6.66667	10.88662	0.552	-30.3866	17.0532
			2.5	0	10.88662	1	-23.7199	23.7199
			4	-6.66667	10.88662	0.552	-30.3866	17.0532
		4	0	-26.66667*	10.88662	0.031	-50.3866	-2.9468
			1.5	-13.33333	10.88662	0.244	-37.0532	10.3866
			2	0	10.88662	1	-23.7199	23.7199
			2.5	6.66667	10.88662	0.552	-17.0532	30.3866
			3	6.66667	10.88662	0.552	-17.0532	30.3866
Pupation160	LSD	0	1.5	33.33333*	12.17161	0.018	6.8137	59.853
			2	53.33333*	12.17161	0.001	26.8137	79.853
			2.5	53.33333*	12.17161	0.001	26.8137	79.853
			3	66.66667*	12.17161	0	40.147	93.1863
			4	60.00000*	12.17161	0	33.4803	86.5197
		1.5	0	-33.33333*	12.17161	0.018	-59.853	-6.8137
			2	20	12.17161	0.126	-6.5197	46.5197
			2.5	20	12.17161	0.126	-6.5197	46.5197

			3	33.33333*	12.17161	0.018	6.8137	59.853
			4	26.66667*	12.17161	0.049	0.147	53.1863
		2	0	-53.33333*	12.17161	0.001	-79.853	-26.8137
			1.5	-20	12.17161	0.126	-46.5197	6.5197
			2.5	0	12.17161	1	-26.5197	26.5197
			3	13.33333	12.17161	0.295	-13.1863	39.853
			4	6.66667	12.17161	0.594	-19.853	33.1863
		2.5	0	-53.33333*	12.17161	0.001	-79.853	-26.8137
			1.5	-20	12.17161	0.126	-46.5197	6.5197
			2	0	12.17161	1	-26.5197	26.5197
			3	13.33333	12.17161	0.295	-13.1863	39.853
			4	6.66667	12.17161	0.594	-19.853	33.1863
		3	0	-66.66667*	12.17161	0	-93.1863	-40.147
			1.5	-33.33333*	12.17161	0.018	-59.853	-6.8137
			2	-13.33333	12.17161	0.295	-39.853	13.1863
			2.5	-13.33333	12.17161	0.295	-39.853	13.1863
			4	-6.66667	12.17161	0.594	-33.1863	19.853
		4	0	-60.00000*	12.17161	0	-86.5197	-33.4803
			1.5	-26.66667*	12.17161	0.049	-53.1863	-0.147
			2	-6.66667	12.17161	0.594	-33.1863	19.853
			2.5	-6.66667	12.17161	0.594	-33.1863	19.853
			3	6.66667	12.17161	0.594	-19.853	33.1863
Pupation168	LSD	0	1.5	13.33333	13.87777	0.356	-16.9037	43.5704
			2	46.66667*	13.87777	0.006	16.4296	76.9037
			2.5	46.66667*	13.87777	0.006	16.4296	76.9037
			3	60.00000*	13.87777	0.001	29.7629	90.2371
			4	53.33333*	13.87777	0.002	23.0963	83.5704
		1.5	0	-13.33333	13.87777	0.356	-43.5704	16.9037
			2	33.33333*	13.87777	0.033	3.0963	63.5704
			2.5	33.33333*	13.87777	0.033	3.0963	63.5704
			3	46.66667*	13.87777	0.006	16.4296	76.9037
			4	40.00000*	13.87777	0.014	9.7629	70.2371
		2	0	-46.66667*	13.87777	0.006	-76.9037	-16.4296
			1.5	-33.33333*	13.87777	0.033	-63.5704	-3.0963
			2.5	0	13.87777	1	-30.2371	30.2371
			3	13.33333	13.87777	0.356	-16.9037	43.5704
			4	6.66667	13.87777	0.64	-23.5704	36.9037
		2.5	0	-46.66667*	13.87777	0.006	-76.9037	-16.4296
			1.5	-33.33333*	13.87777	0.033	-63.5704	-3.0963
			2	0	13.87777	1	-30.2371	30.2371
			3	13.33333	13.87777	0.356	-16.9037	43.5704
			4	6.66667	13.87777	0.64	-23.5704	36.9037
		3	0	-60.00000*	13.87777	0.001	-90.2371	-29.7629
			1.5	-46.66667*	13.87777	0.006	-76.9037	-16.4296
			2	-13.33333	13.87777	0.356	-43.5704	16.9037
			2.5	-13.33333	13.87777	0.356	-43.5704	16.9037
			4	-6.66667	13.87777	0.64	-36.9037	23.5704
		4	0	-53.33333*	13.87777	0.002	-83.5704	-23.0963
			1.5	-40.00000*	13.87777	0.014	-70.2371	-9.7629
			2	-6.66667	13.87777	0.64	-36.9037	23.5704
			2.5	-6.66667	13.87777	0.64	-36.9037	23.5704
			3	6.66667	13.87777	0.64	-23.5704	36.9037

Pupation192	LSD	0	1.5	20	13.33333	0.159	-9.0508	49.0508
			2	46.66667*	13.33333	0.004	17.6158	75.7175
			2.5	46.66667*	13.33333	0.004	17.6158	75.7175
			3	60.00000*	13.33333	0.001	30.9492	89.0508
			4	66.66667*	13.33333	0	37.6158	95.7175
		1.5	0	-20	13.33333	0.159	-49.0508	9.0508
			2	26.66667	13.33333	0.069	-2.3842	55.7175
			2.5	26.66667	13.33333	0.069	-2.3842	55.7175
			3	40.00000*	13.33333	0.011	10.9492	69.0508
			4	46.66667*	13.33333	0.004	17.6158	75.7175
		2	0	-46.66667*	13.33333	0.004	-75.7175	-17.6158
			1.5	-26.66667	13.33333	0.069	-55.7175	2.3842
			2.5	0	13.33333	1	-29.0508	29.0508
			3	13.33333	13.33333	0.337	-15.7175	42.3842
			4	20	13.33333	0.159	-9.0508	49.0508
		2.5	0	-46.66667*	13.33333	0.004	-75.7175	-17.6158
			1.5	-26.66667	13.33333	0.069	-55.7175	2.3842
			2	0	13.33333	1	-29.0508	29.0508
			3	13.33333	13.33333	0.337	-15.7175	42.3842
			4	20	13.33333	0.159	-9.0508	49.0508
		3	0	-60.00000*	13.33333	0.001	-89.0508	-30.9492
			1.5	-40.00000*	13.33333	0.011	-69.0508	-10.9492
			2	-13.33333	13.33333	0.337	-42.3842	15.7175
			2.5	-13.33333	13.33333	0.337	-42.3842	15.7175
			4	6.66667	13.33333	0.626	-22.3842	35.7175
		4	0	-66.66667*	13.33333	0	-95.7175	-37.6158
			1.5	-46.66667*	13.33333	0.004	-75.7175	-17.6158
			2	-20	13.33333	0.159	-49.0508	9.0508
			2.5	-20	13.33333	0.159	-49.0508	9.0508
			3	-6.66667	13.33333	0.626	-35.7175	22.3842
Pupation216	LSD	0	1.5	6.66667	14.90712	0.663	-25.8132	39.1465
			2	40.00000*	14.90712	0.02	7.5202	72.4798
			2.5	40.00000*	14.90712	0.02	7.5202	72.4798
			3	53.33333*	14.90712	0.004	20.8535	85.8132
			4	66.66667*	14.90712	0.001	34.1868	99.1465
		1.5	0	-6.66667	14.90712	0.663	-39.1465	25.8132
			2	33.33333*	14.90712	0.045	0.8535	65.8132
			2.5	33.33333*	14.90712	0.045	0.8535	65.8132
			3	46.66667*	14.90712	0.009	14.1868	79.1465
			4	60.00000*	14.90712	0.002	27.5202	92.4798
		2	0	-40.00000*	14.90712	0.02	-72.4798	-7.5202
			1.5	-33.33333*	14.90712	0.045	-65.8132	-0.8535
			2.5	0	14.90712	1	-32.4798	32.4798
			3	13.33333	14.90712	0.389	-19.1465	45.8132
			4	26.66667	14.90712	0.099	-5.8132	59.1465
		2.5	0	-40.00000*	14.90712	0.02	-72.4798	-7.5202
			1.5	-33.33333*	14.90712	0.045	-65.8132	-0.8535
			2	0	14.90712	1	-32.4798	32.4798
			3	13.33333	14.90712	0.389	-19.1465	45.8132
			4	26.66667	14.90712	0.099	-5.8132	59.1465
		3	0	-53.33333*	14.90712	0.004	-85.8132	-20.8535
			1.5	-46.66667*	14.90712	0.009	-79.1465	-14.1868

			2	-13.33333	14.90712	0.389	-45.8132	19.1465
			2.5	-13.33333	14.90712	0.389	-45.8132	19.1465
			4	13.33333	14.90712	0.389	-19.1465	45.8132
		4	0	-66.66667*	14.90712	0.001	-99.1465	-34.1868
			1.5	-60.00000*	14.90712	0.002	-92.4798	-27.5202
			2	-26.66667	14.90712	0.099	-59.1465	5.8132
			2.5	-26.66667	14.90712	0.099	-59.1465	5.8132
			3	-13.33333	14.90712	0.389	-45.8132	19.1465
Pupation240	LSD	0	1.5	6.66667	13.33333	0.626	-22.3842	35.7175
			2	33.33333*	13.33333	0.028	4.2825	62.3842
			2.5	40.00000*	13.33333	0.011	10.9492	69.0508
			3	46.66667*	13.33333	0.004	17.6158	75.7175
			4	60.00000*	13.33333	0.001	30.9492	89.0508
		1.5	0	-6.66667	13.33333	0.626	-35.7175	22.3842
			2	26.66667	13.33333	0.069	-2.3842	55.7175
			2.5	33.33333*	13.33333	0.028	4.2825	62.3842
			3	40.00000*	13.33333	0.011	10.9492	69.0508
			4	53.33333*	13.33333	0.002	24.2825	82.3842
		2	0	-33.33333*	13.33333	0.028	-62.3842	-4.2825
			1.5	-26.66667	13.33333	0.069	-55.7175	2.3842
			2.5	6.66667	13.33333	0.626	-22.3842	35.7175
			3	13.33333	13.33333	0.337	-15.7175	42.3842
			4	26.66667	13.33333	0.069	-2.3842	55.7175
		2.5	0	-40.00000*	13.33333	0.011	-69.0508	-10.9492
			1.5	-33.33333*	13.33333	0.028	-62.3842	-4.2825
			2	-6.66667	13.33333	0.626	-35.7175	22.3842
			3	6.66667	13.33333	0.626	-22.3842	35.7175
			4	20	13.33333	0.159	-9.0508	49.0508
		3	0	-46.66667*	13.33333	0.004	-75.7175	-17.6158
			1.5	-40.00000*	13.33333	0.011	-69.0508	-10.9492
			2	-13.33333	13.33333	0.337	-42.3842	15.7175
			2.5	-6.66667	13.33333	0.626	-35.7175	22.3842
			4	13.33333	13.33333	0.337	-15.7175	42.3842
		4	0	-60.00000*	13.33333	0.001	-89.0508	-30.9492
			1.5	-53.33333*	13.33333	0.002	-82.3842	-24.2825
			2	-26.66667	13.33333	0.069	-55.7175	2.3842
			2.5	-20	13.33333	0.159	-49.0508	9.0508
			3	-13.33333	13.33333	0.337	-42.3842	15.7175

* The mean difference is significant at the 0.05 level.

Table S11. ANOVA results of duration of pupation, time of pupation onset, and time of pupation end.

		Sum of Squares	df	Mean Square	F	Sig.
Duration of pupation	Between Groups	1412.444	5	282.489	0.404	0.837
	Within Groups	8394.667	12	699.556		
	Total	9807.111	17			
Time of pupation onset	Between Groups	2236.444	5	447.289	2.132	0.131
	Within Groups	2517.333	12	209.778		
	Total	4753.778	17			
Time of pupation end	Between Groups	3488	5	697.6	0.735	0.611
	Within Groups	11392	12	949.333		
	Total	14880	17			

Table S12. Post hoc tests results of duration of pupation, time of pupation onset, and time of pupation end.

**Multiple Comparisons**								
**Dependent Variable**		(I) Concentration	(J) Concentration	Mean Difference (I-J)	Std. Error	Sig.	95% Confidence Interval	
							Lower Bound	Upper Bound
Duration	LSD	0	1.5	-18.66667	21.59561	0.404	-65.7195	28.3861
			2	-21.33333	21.59561	0.343	-68.3861	25.7195
			2.5	-12	21.59561	0.589	-59.0528	35.0528
			3	-8	21.59561	0.718	-55.0528	39.0528
			4	2.66667	21.59561	0.904	-44.3861	49.7195
		1.5	0	18.66667	21.59561	0.404	-28.3861	65.7195
			2	-2.66667	21.59561	0.904	-49.7195	44.3861
			2.5	6.66667	21.59561	0.763	-40.3861	53.7195
			3	10.66667	21.59561	0.63	-36.3861	57.7195
			4	21.33333	21.59561	0.343	-25.7195	68.3861
		2	0	21.33333	21.59561	0.343	-25.7195	68.3861
			1.5	2.66667	21.59561	0.904	-44.3861	49.7195
			2.5	9.33333	21.59561	0.673	-37.7195	56.3861
			3	13.33333	21.59561	0.549	-33.7195	60.3861
			4	24	21.59561	0.288	-23.0528	71.0528
		2.5	0	12	21.59561	0.589	-35.0528	59.0528
			1.5	-6.66667	21.59561	0.763	-53.7195	40.3861
			2	-9.33333	21.59561	0.673	-56.3861	37.7195
			3	4	21.59561	0.856	-43.0528	51.0528
			4	14.66667	21.59561	0.51	-32.3861	61.7195
		3	0	8	21.59561	0.718	-39.0528	55.0528
			1.5	-10.66667	21.59561	0.63	-57.7195	36.3861
			2	-13.33333	21.59561	0.549	-60.3861	33.7195
			2.5	-4	21.59561	0.856	-51.0528	43.0528
			4	10.66667	21.59561	0.63	-36.3861	57.7195
		4	0	-2.66667	21.59561	0.904	-49.7195	44.3861
			1.5	-21.33333	21.59561	0.343	-68.3861	25.7195
			2	-24	21.59561	0.288	-71.0528	23.0528
			2.5	-14.66667	21.59561	0.51	-61.7195	32.3861
			3	-10.66667	21.59561	0.63	-57.7195	36.3861
Onset	LSD	0	1.5	-8	11.8259	0.512	-33.7664	17.7664
			2	-13.33333	11.8259	0.282	-39.0998	12.4331
			2.5	-24	11.8259	0.065	-49.7664	1.7664
			3	-34.66667*	11.8259	0.013	-60.4331	-8.9002
			4	-18.66667	11.8259	0.14	-44.4331	7.0998
		1.5	0	8	11.8259	0.512	-17.7664	33.7664
			2	-5.33333	11.8259	0.66	-31.0998	20.4331
			2.5	-16	11.8259	0.201	-41.7664	9.7664
			3	-26.66667*	11.8259	0.044	-52.4331	-0.9002
			4	-10.66667	11.8259	0.385	-36.4331	15.0998
		2	0	13.33333	11.8259	0.282	-12.4331	39.0998
			1.5	5.33333	11.8259	0.66	-20.4331	31.0998
			2.5	-10.66667	11.8259	0.385	-36.4331	15.0998
			3	-21.33333	11.8259	0.096	-47.0998	4.4331
			4	-5.33333	11.8259	0.66	-31.0998	20.4331

		2.5	0	24	11.8259	0.065	-1.7664	49.7664
			1.5	16	11.8259	0.201	-9.7664	41.7664
			2	10.66667	11.8259	0.385	-15.0998	36.4331
			3	-10.66667	11.8259	0.385	-36.4331	15.0998
			4	5.33333	11.8259	0.66	-20.4331	31.0998
		3	0	34.66667*	11.8259	0.013	8.9002	60.4331
			1.5	26.66667*	11.8259	0.044	0.9002	52.4331
			2	21.33333	11.8259	0.096	-4.4331	47.0998
			2.5	10.66667	11.8259	0.385	-15.0998	36.4331
			4	16	11.8259	0.201	-9.7664	41.7664
		4	0	18.66667	11.8259	0.14	-7.0998	44.4331
			1.5	10.66667	11.8259	0.385	-15.0998	36.4331
			2	5.33333	11.8259	0.66	-20.4331	31.0998
			2.5	-5.33333	11.8259	0.66	-31.0998	20.4331
			3	-16	11.8259	0.201	-41.7664	9.7664
End	LSD	0	1.5	-26.66667	25.15728	0.31	-81.4797	28.1463
			2	-34.66667	25.15728	0.193	-89.4797	20.1463
			2.5	-16	25.15728	0.537	-70.813	38.813
			3	-42.66667	25.15728	0.116	-97.4797	12.1463
			4	-16	25.15728	0.537	-70.813	38.813
		1.5	0	26.66667	25.15728	0.31	-28.1463	81.4797
			2	-8	25.15728	0.756	-62.813	46.813
			2.5	10.66667	25.15728	0.679	-44.1463	65.4797
			3	-16	25.15728	0.537	-70.813	38.813
			4	10.66667	25.15728	0.679	-44.1463	65.4797
		2	0	34.66667	25.15728	0.193	-20.1463	89.4797
			1.5	8	25.15728	0.756	-46.813	62.813
			2.5	18.66667	25.15728	0.472	-36.1463	73.4797
			3	-8	25.15728	0.756	-62.813	46.813
			4	18.66667	25.15728	0.472	-36.1463	73.4797
		2.5	0	16	25.15728	0.537	-38.813	70.813
			1.5	-10.66667	25.15728	0.679	-65.4797	44.1463
			2	-18.66667	25.15728	0.472	-73.4797	36.1463
			3	-26.66667	25.15728	0.31	-81.4797	28.1463
			4	0	25.15728	1	-54.813	54.813
		3	0	42.66667	25.15728	0.116	-12.1463	97.4797
			1.5	16	25.15728	0.537	-38.813	70.813
			2	8	25.15728	0.756	-46.813	62.813
			2.5	26.66667	25.15728	0.31	-28.1463	81.4797
			4	26.66667	25.15728	0.31	-28.1463	81.4797
		4	0	16	25.15728	0.537	-38.813	70.813
			1.5	-10.66667	25.15728	0.679	-65.4797	44.1463
			2	-18.66667	25.15728	0.472	-73.4797	36.1463
			2.5	0	25.15728	1	-54.813	54.813
			3	-26.66667	25.15728	0.31	-81.4797	28.1463

* The mean difference is significant at the 0.05 level.

Table S13. ANOVA results of gene expression levels of *ecr*, *e74*, and *usp*.

		Sum of Squares	df	Mean Square	F	Sig.
ecr	Between Groups	82.882	7	11.84	70.862	0
	Within Groups	2.673	16	0.167		
	Total	85.555	23			
e74	Between Groups	143.526	7	20.504	128.918	0
	Within Groups	2.545	16	0.159		
	Total	146.07	23			
usp	Between Groups	63.289	7	9.041	79.528	0
	Within Groups	1.819	16	0.114		
	Total	65.108	23			

Table S14. Post hoc tests results of gene expression levels of *ecr*, *e74*, and *usp*.

Multiple Comparisons								
**Dependent Variable**		(I) Group	(J) Group	Mean Difference (I-J)	Std. Error	Sig.	95% Confidence Interval	
							Lower Bound	Upper Bound
ecr	LSD	Control0	Exposure0	0	0.33375	1	-0.7075	0.7075
			Control72	-2.68257*	0.33375	0	-3.3901	-1.975
			Exposure72	-2.97585*	0.33375	0	-3.6834	-2.2683
			Control120	-5.10145*	0.33375	0	-5.809	-4.3939
			Exposure120	-3.12049*	0.33375	0	-3.828	-2.413
			Control144	-5.30743*	0.33375	0	-6.015	-4.5999
			Exposure144	-2.21721*	0.33375	0	-2.9247	-1.5097
		Exposure0	Control0	0	0.33375	1	-0.7075	0.7075
			Control72	-2.68257*	0.33375	0	-3.3901	-1.975
			Exposure72	-2.97585*	0.33375	0	-3.6834	-2.2683
			Control120	-5.10145*	0.33375	0	-5.809	-4.3939
			Exposure120	-3.12049*	0.33375	0	-3.828	-2.413
			Control144	-5.30743*	0.33375	0	-6.015	-4.5999
			Exposure144	-2.21721*	0.33375	0	-2.9247	-1.5097
		Control72	Control0	2.68257*	0.33375	0	1.975	3.3901
			Exposure0	2.68257*	0.33375	0	1.975	3.3901
			Exposure72	-0.29328	0.33375	0.393	-1.0008	0.4143
			Control120	-2.41888*	0.33375	0	-3.1264	-1.7114
			Exposure120	-0.43792	0.33375	0.208	-1.1454	0.2696
			Control144	-2.62485*	0.33375	0	-3.3324	-1.9173
			Exposure144	0.46537	0.33375	0.182	-0.2422	1.1729
		Exposure72	Control0	2.97585*	0.33375	0	2.2683	3.6834
			Exposure0	2.97585*	0.33375	0	2.2683	3.6834
			Control72	0.29328	0.33375	0.393	-0.4143	1.0008
			Control120	-2.12560*	0.33375	0	-2.8331	-1.4181
			Exposure120	-0.14464	0.33375	0.671	-0.8522	0.5629
			Control144	-2.33158*	0.33375	0	-3.0391	-1.6241
			Exposure144	.75864*	0.33375	0.037	0.0511	1.4662
		Control120	Control0	5.10145*	0.33375	0	4.3939	5.809
			Exposure0	5.10145*	0.33375	0	4.3939	5.809
			Control72	2.41888*	0.33375	0	1.7114	3.1264
			Exposure72	2.12560*	0.33375	0	1.4181	2.8331
			Exposure120	1.98096*	0.33375	0	1.2734	2.6885
			Control144	-0.20597	0.33375	0.546	-0.9135	0.5016
			Exposure144	2.88425*	0.33375	0	2.1767	3.5918
		Exposure120	Control0	3.12049*	0.33375	0	2.413	3.828
			Exposure0	3.12049*	0.33375	0	2.413	3.828
			Control72	0.43792	0.33375	0.208	-0.2696	1.1454
			Exposure72	0.14464	0.33375	0.671	-0.5629	0.8522
			Control120	-1.98096*	0.33375	0	-2.6885	-1.2734
			Control144	-2.18694*	0.33375	0	-2.8945	-1.4794
			Exposure144	.90328*	0.33375	0.016	0.1958	1.6108
		Control144	Control0	5.30743*	0.33375	0	4.5999	6.015
			Exposure0	5.30743*	0.33375	0	4.5999	6.015
			Control72	2.62485*	0.33375	0	1.9173	3.3324
			Exposure72	2.33158*	0.33375	0	1.6241	3.0391

			Control120	0.20597	0.33375	0.546	-0.5016	0.9135
			Exposure120	2.18694*	0.33375	0	1.4794	2.8945
			Exposure144	3.09022*	0.33375	0	2.3827	3.7977
		Exposure144	Control0	2.21721*	0.33375	0	1.5097	2.9247
			Exposure0	2.21721*	0.33375	0	1.5097	2.9247
			Control72	-0.46537	0.33375	0.182	-1.1729	0.2422
			Exposure72	-.75864*	0.33375	0.037	-1.4662	-0.0511
			Control120	-2.88425*	0.33375	0	-3.5918	-2.1767
			Exposure120	-.90328*	0.33375	0.016	-1.6108	-0.1958
			Control144	-3.09022*	0.33375	0	-3.7977	-2.3827
e74	LSD	Control0	Exposure0	0	0.32562	1	-0.6903	0.6903
			Control72	-2.78091*	0.32562	0	-3.4712	-2.0906
			Exposure72	-2.34955*	0.32562	0	-3.0398	-1.6593
			Control120	-4.74466*	0.32562	0	-5.435	-4.0544
			Exposure120	-5.33098*	0.32562	0	-6.0213	-4.6407
			Control144	-7.42031*	0.32562	0	-8.1106	-6.73
			Exposure144	-1.82533*	0.32562	0	-2.5156	-1.135
		Exposure0	Control0	0	0.32562	1	-0.6903	0.6903
			Control72	-2.78091*	0.32562	0	-3.4712	-2.0906
			Exposure72	-2.34955*	0.32562	0	-3.0398	-1.6593
			Control120	-4.74466*	0.32562	0	-5.435	-4.0544
			Exposure120	-5.33098*	0.32562	0	-6.0213	-4.6407
			Control144	-7.42031*	0.32562	0	-8.1106	-6.73
			Exposure144	-1.82533*	0.32562	0	-2.5156	-1.135
		Control72	Control0	2.78091*	0.32562	0	2.0906	3.4712
			Exposure0	2.78091*	0.32562	0	2.0906	3.4712
			Exposure72	0.43137	0.32562	0.204	-0.2589	1.1217
			Control120	-1.96375*	0.32562	0	-2.654	-1.2735
			Exposure120	-2.55007*	0.32562	0	-3.2404	-1.8598
			Control144	-4.63940*	0.32562	0	-5.3297	-3.9491
			Exposure144	.95558*	0.32562	0.01	0.2653	1.6459
		Exposure72	Control0	2.34955*	0.32562	0	1.6593	3.0398
			Exposure0	2.34955*	0.32562	0	1.6593	3.0398
			Control72	-0.43137	0.32562	0.204	-1.1217	0.2589
			Control120	-2.39512*	0.32562	0	-3.0854	-1.7048
			Exposure120	-2.98144*	0.32562	0	-3.6717	-2.2911
			Control144	-5.07076*	0.32562	0	-5.7611	-4.3805
			Exposure144	0.52422	0.32562	0.127	-0.1661	1.2145
		Control120	Control0	4.74466*	0.32562	0	4.0544	5.435
			Exposure0	4.74466*	0.32562	0	4.0544	5.435
			Control72	1.96375*	0.32562	0	1.2735	2.654
			Exposure72	2.39512*	0.32562	0	1.7048	3.0854
			Exposure120	-0.58632	0.32562	0.091	-1.2766	0.104
			Control144	-2.67565*	0.32562	0	-3.3659	-1.9854
			Exposure144	2.91933*	0.32562	0	2.229	3.6096
		Exposure120	Control0	5.33098*	0.32562	0	4.6407	6.0213
			Exposure0	5.33098*	0.32562	0	4.6407	6.0213
			Control72	2.55007*	0.32562	0	1.8598	3.2404
			Exposure72	2.98144*	0.32562	0	2.2911	3.6717
			Control120	0.58632	0.32562	0.091	-0.104	1.2766
			Control144	-2.08933*	0.32562	0	-2.7796	-1.399
			Exposure144	3.50565*	0.32562	0	2.8154	4.1959

		Control144	Control0	7.42031*	0.32562	0	6.73	8.1106
			Exposure0	7.42031*	0.32562	0	6.73	8.1106
			Control72	4.63940*	0.32562	0	3.9491	5.3297
			Exposure72	5.07076*	0.32562	0	4.3805	5.7611
			Control120	2.67565*	0.32562	0	1.9854	3.3659
			Exposure120	2.08933*	0.32562	0	1.399	2.7796
			Exposure144	5.59498*	0.32562	0	4.9047	6.2853
		Exposure144	Control0	1.82533*	0.32562	0	1.135	2.5156
			Exposure0	1.82533*	0.32562	0	1.135	2.5156
			Control72	-.95558*	0.32562	0.01	-1.6459	-0.2653
			Exposure72	-0.52422	0.32562	0.127	-1.2145	0.1661
			Control120	-2.91933*	0.32562	0	-3.6096	-2.229
			Exposure120	-3.50565*	0.32562	0	-4.1959	-2.8154
			Control144	-5.59498*	0.32562	0	-6.2853	-4.9047
usp	LSD	Control0	Exposure0	0	0.2753	1	-0.5836	0.5836
			Control72	-1.59032*	0.2753	0	-2.1739	-1.0067
			Exposure72	-4.08703*	0.2753	0	-4.6706	-3.5034
			Control120	-4.56994*	0.2753	0	-5.1536	-3.9863
			Exposure120	-2.24850*	0.2753	0	-2.8321	-1.6649
			Control144	-.84696*	0.2753	0.007	-1.4306	-0.2633
			Exposure144	-1.05170*	0.2753	0.002	-1.6353	-0.4681
		Exposure0	Control0	0	0.2753	1	-0.5836	0.5836
			Control72	-1.59032*	0.2753	0	-2.1739	-1.0067
			Exposure72	-4.08703*	0.2753	0	-4.6706	-3.5034
			Control120	-4.56994*	0.2753	0	-5.1536	-3.9863
			Exposure120	-2.24850*	0.2753	0	-2.8321	-1.6649
			Control144	-.84696*	0.2753	0.007	-1.4306	-0.2633
			Exposure144	-1.05170*	0.2753	0.002	-1.6353	-0.4681
		Control72	Control0	1.59032*	0.2753	0	1.0067	2.1739
			Exposure0	1.59032*	0.2753	0	1.0067	2.1739
			Exposure72	-2.49671*	0.2753	0	-3.0803	-1.9131
			Control120	-2.97962*	0.2753	0	-3.5632	-2.396
			Exposure120	-.65818*	0.2753	0.029	-1.2418	-0.0746
			Control144	.74336*	0.2753	0.016	0.1597	1.327
			Exposure144	0.53862	0.2753	0.068	-0.045	1.1222
		Exposure72	Control0	4.08703*	0.2753	0	3.5034	4.6706
			Exposure0	4.08703*	0.2753	0	3.5034	4.6706
			Control72	2.49671*	0.2753	0	1.9131	3.0803
			Control120	-0.48291	0.2753	0.099	-1.0665	0.1007
			Exposure120	1.83853*	0.2753	0	1.2549	2.4221
			Control144	3.24007*	0.2753	0	2.6565	3.8237
			Exposure144	3.03532*	0.2753	0	2.4517	3.6189
		Control120	Control0	4.56994*	0.2753	0	3.9863	5.1536
			Exposure0	4.56994*	0.2753	0	3.9863	5.1536
			Control72	2.97962*	0.2753	0	2.396	3.5632
			Exposure72	0.48291	0.2753	0.099	-0.1007	1.0665
			Exposure120	2.32144*	0.2753	0	1.7378	2.905
			Control144	3.72298*	0.2753	0	3.1394	4.3066
			Exposure144	3.51823*	0.2753	0	2.9346	4.1018
		Exposure120	Control0	2.24850*	0.2753	0	1.6649	2.8321
			Exposure0	2.24850*	0.2753	0	1.6649	2.8321
			Control72	.65818*	0.2753	0.029	0.0746	1.2418

	Exposure72	-1.83853*	0.2753	0	-2.4221	-1.2549
	Control120	-2.32144*	0.2753	0	-2.905	-1.7378
	Control144	1.40154*	0.2753	0	0.8179	1.9852
	Exposure144	1.19680*	0.2753	0	0.6132	1.7804
Control144	Control0	.84696*	0.2753	0.007	0.2633	1.4306
	Exposure0	.84696*	0.2753	0.007	0.2633	1.4306
	Control72	-.74336*	0.2753	0.016	-1.327	-0.1597
	Exposure72	-3.24007*	0.2753	0	-3.8237	-2.6565
	Control120	-3.72298*	0.2753	0	-4.3066	-3.1394
	Exposure120	-1.40154*	0.2753	0	-1.9852	-0.8179
	Exposure144	-0.20474	0.2753	0.468	-0.7884	0.3789
Exposure144	Control0	1.05170*	0.2753	0.002	0.4681	1.6353
	Exposure0	1.05170*	0.2753	0.002	0.4681	1.6353
	Control72	-0.53862	0.2753	0.068	-1.1222	0.045
	Exposure72	-3.03532*	0.2753	0	-3.6189	-2.4517
	Control120	-3.51823*	0.2753	0	-4.1018	-2.9346
	Exposure120	-1.19680*	0.2753	0	-1.7804	-0.6132
	Control144	0.20474	0.2753	0.468	-0.3789	0.7884

* The mean difference is significant at the 0.05 level.

Table S15. ANOVA results of area of imaginal discs.

	Sum of Squares	df	Mean Square	F	Sig.
Between Groups	250969631.4	2	125484815.7	8.973	0.016
Within Groups	83907956.91	6	13984659.49		
Total	334877588.3	8			

Table S16. Post hoc tests results of area of imaginal discs.

**Multiple Comparisons**							
**Dependent Variable: Area**							
	(I) Concentration	(J) Concentration	Mean Difference (I-J)	Std. Error	Sig.	95% Confidence Interval	
						Lower Bound	Upper Bound
LSD	0	2	6535.81367	3053.37622	0.076	-935.5288	14007.1561
		4	12934.71450*	3053.37622	0.005	5463.372	20406.057
	2	0	-6535.8137	3053.37622	0.076	-14007.156	935.5288
		4	6398.90083	3053.37622	0.081	-1072.4416	13870.2433
	4	0	-12934.71450*	3053.37622	0.005	-20406.057	-5463.372
		2	-6398.9008	3053.37622	0.081	-13870.243	1072.4416

* The mean difference is significant at the 0.05 level.

Table S17. ANOVA results of area of yolk granules.

	Sum of Squares	df	Mean Square	F	Sig.
Between Groups	870.364	3	290.121	40.675	0
Within Groups	827.393	116	7.133		
Total	1697.757	119			

Table S18. Post hoc tests results of area of yolk granules.

Multiple Comparisons							
**Dependent Variable: YolkArea**							
	(I) Concentration	(J) Concentration	Mean Difference (I-J)	Std. Error	Sig.	95% Confidence Interval	
						Lower Bound	Upper Bound
LSD	0	0.5	1.53333*	0.68957	0.028	0.1675	2.8991
		1	4.97267*	0.68957	0	3.6069	6.3385
		2	6.79367*	0.68957	0	5.4279	8.1595
	0.5	0	-1.53333*	0.68957	0.028	-2.8991	-0.1675
		1	3.43933*	0.68957	0	2.0735	4.8051
		2	5.26033*	0.68957	0	3.8945	6.6261
	1	0	-4.97267*	0.68957	0	-6.3385	-3.6069
		0.5	-3.43933*	0.68957	0	-4.8051	-2.0735
		2	1.82100*	0.68957	0.009	0.4552	3.1868
	2	0	-6.79367*	0.68957	0	-8.1595	-5.4279
		0.5	-5.26033*	0.68957	0	-6.6261	-3.8945
		1	-1.82100*	0.68957	0.009	-3.1868	-0.4552

* The mean difference is significant at the 0.05 level.

Table S19. ANOVA results of area of spermatids.

	Sum of Squares	df	Mean Square	F	Sig.
Between Groups	3158.298	3	1052.766	15.697	0
Within Groups	7779.97	116	67.069		
Total	10938.268	119			

Table S20. Post hoc tests results of area of spermatids.

**Multiple Comparisons**							
	**Dependent Variable: SpermatidArea**						
	(I) Concentration	(J) Concentration	Mean Difference (I-J)	Std. Error	Sig.	95% Confidence Interval	
						Lower Bound	Upper Bound
LSD	0	0.5	-0.27567	2.11453	0.897	-4.4638	3.9124
		1	-3.576	2.11453	0.093	-7.7641	0.6121
		2	-12.67733*	2.11453	0	-16.8654	-8.4892
	0.5	0	0.27567	2.11453	0.897	-3.9124	4.4638
		1	-3.30033	2.11453	0.121	-7.4884	0.8878
		2	-12.40167*	2.11453	0	-16.5898	-8.2136
	1	0	3.576	2.11453	0.093	-0.6121	7.7641
		0.5	3.30033	2.11453	0.121	-0.8878	7.4884
		2	-9.10133*	2.11453	0	-13.2894	-4.9132
	2	0	12.67733*	2.11453	0	8.4892	16.8654
		0.5	12.40167*	2.11453	0	8.2136	16.5898
		1	9.10133*	2.11453	0	4.9132	13.2894
	* The mean difference is significant at the 0.05 level.						

Table S21. ANOVA results of spawning rate of treated females × untreated males.

	**Sum of Squares**	**df**	**Mean Square**	**F**	**Sig.**
Between Groups	157.482	3	52.494	1.054	0.42
Within Groups	398.338	8	49.792		
Total	555.82	11			

Table S22. Post hoc tests results of spawning rate of treated females × untreated males.

Multiple Comparisons							
**Dependent Variable: SpawningFemale**							
	(I) Concentration	(J) Concentration	Mean Difference (I-J)	Std. Error	Sig.	95% Confidence Interval	
						Lower Bound	Upper Bound
LSD	0	0.5	8.00977	5.7615	0.202	-5.2763	21.2958
		1	-1.48352	5.7615	0.803	-14.7695	11.8025
		2	1.50794	5.7615	0.8	-11.7781	14.794
	0.5	0	-8.00977	5.7615	0.202	-21.2958	5.2763
		1	-9.49328	5.7615	0.138	-22.7793	3.7927
		2	-6.50183	5.7615	0.292	-19.7879	6.7842
	1	0	1.48352	5.7615	0.803	-11.8025	14.7695
		0.5	9.49328	5.7615	0.138	-3.7927	22.7793
		2	2.99145	5.7615	0.618	-10.2946	16.2775
	2	0	-1.50794	5.7615	0.8	-14.794	11.7781
		0.5	6.50183	5.7615	0.292	-6.7842	19.7879
		1	-2.99145	5.7615	0.618	-16.2775	10.2946

Table S23. ANOVA results of spawning rate of treated males × untreated females.

	**Sum of Squares**	**df**	**Mean Square**	**F**	**Sig.**
Between Groups	2056.71	3	685.57	13.718	0.002
Within Groups	399.804	8	49.975		
Total	2456.514	11			

Table S24. Post hoc tests results of spawning rate of treated males × untreated females.

**Multiple Comparisons**							
	**Dependent Variable: SpawningMale**						
	(I) Concentration	(J) Concentration	Mean Difference (I-J)	Std. Error	Sig.	95% Confidence Interval	
						Lower Bound	Upper Bound
LSD	0	0.5	-1.13553	5.77209	0.849	-14.446	12.1749
		1	16.88034*	5.77209	0.019	3.5699	30.1908
		2	30.59829*	5.77209	0.001	17.2878	43.9087
	0.5	0	1.13553	5.77209	0.849	-12.1749	14.446
		1	18.01587*	5.77209	0.014	4.7054	31.3263
		2	31.73382*	5.77209	0.001	18.4234	45.0443
	1	0	-16.88034*	5.77209	0.019	-30.1908	-3.5699
		0.5	-18.01587*	5.77209	0.014	-31.3263	-4.7054
		2	13.71795*	5.77209	0.045	0.4075	27.0284
	2	0	-30.59829*	5.77209	0.001	-43.9087	-17.2878
		0.5	-31.73382*	5.77209	0.001	-45.0443	-18.4234
		1	-13.71795*	5.77209	0.045	-27.0284	-0.4075

* The mean difference is significant at the 0.05 level.

Table S25. ANOVA results of gene expression levels of *ecr*, *kr-h1*, *foxo*, *inr*, *pdk*, *akt*, and *vg* in females.

		**Sum of Squares**	**df**	**Mean Square**	**F**	**Sig.**
ecr	Between Groups	2.171	3	0.724	7.718	0.01
	Within Groups	0.75	8	0.094		
	Total	2.922	11			
kr-h1	Between Groups	1.235	3	0.412	3.475	0.071
	Within Groups	0.948	8	0.118		
	Total	2.183	11			
foxo	Between Groups	1.935	3	0.645	9.886	0.005
	Within Groups	0.522	8	0.065		
	Total	2.457	11			
inr	Between Groups	3.449	3	1.15	7.541	0.01
	Within Groups	1.22	8	0.152		
	Total	4.669	11			
pdk	Between Groups	1.614	3	0.538	4.438	0.041
	Within Groups	0.97	8	0.121		
	Total	2.584	11			
akt	Between Groups	1.192	3	0.397	4.787	0.034
	Within Groups	0.664	8	0.083		
	Total	1.857	11			
vg	Between Groups	1.47	3	0.49	9.014	0.006
	Within Groups	0.435	8	0.054		
	Total	1.905	11			

Table S26. Post hoc tests results of gene expression levels of *ecr*, *kr-h1*, *foxo*, *inr*, *pdk*, *akt*, and *vg* in females.

**Multiple Comparisons**								
**Dependent Variable**		(I) Concentration	(J) Concentration	Mean Difference (I-J)	Std. Error	Sig.	95% Confidence Interval	
							Lower Bound	Upper Bound
ecr	LSD	0	0.5	-0.45	0.25003	0.11	-1.0266	0.1266
			1	-0.13	0.25003	0.617	-0.7066	0.4466
			2	-1.10000*	0.25003	0.002	-1.6766	-0.5234
		0.5	0	0.45	0.25003	0.11	-0.1266	1.0266
			1	0.32	0.25003	0.236	-0.2566	0.8966
			2	-.65000*	0.25003	0.032	-1.2266	-0.0734
		1	0	0.13	0.25003	0.617	-0.4466	0.7066
			0.5	-0.32	0.25003	0.236	-0.8966	0.2566
			2	-.97000*	0.25003	0.005	-1.5466	-0.3934
		2	0	1.10000*	0.25003	0.002	0.5234	1.6766
			0.5	.65000*	0.25003	0.032	0.0734	1.2266
			1	.97000*	0.25003	0.005	0.3934	1.5466
Kr-h1	LSD	0	0.5	-0.5	0.28104	0.113	-1.1481	0.1481
			1	-.80000*	0.28104	0.022	-1.4481	-0.1519
			2	-.77000*	0.28104	0.025	-1.4181	-0.1219
		0.5	0	0.5	0.28104	0.113	-0.1481	1.1481
			1	-0.3	0.28104	0.317	-0.9481	0.3481
			2	-0.27	0.28104	0.365	-0.9181	0.3781
		1	0	.80000*	0.28104	0.022	0.1519	1.4481
			0.5	0.3	0.28104	0.317	-0.3481	0.9481
			2	0.03	0.28104	0.918	-0.6181	0.6781
		2	0	.77000*	0.28104	0.025	0.1219	1.4181
			0.5	0.27	0.28104	0.365	-0.3781	0.9181
			1	-0.03	0.28104	0.918	-0.6781	0.6181
foxo	LSD	0	0.5	-.69000*	0.20857	0.011	-1.171	-0.209
			1	-.75000*	0.20857	0.007	-1.231	-0.269
			2	-1.11000*	0.20857	0.001	-1.591	-0.629
		0.5	0	.69000*	0.20857	0.011	0.209	1.171
			1	-0.06	0.20857	0.781	-0.541	0.421
			2	-0.42	0.20857	0.079	-0.901	0.061
		1	0	.75000*	0.20857	0.007	0.269	1.231
			0.5	0.06	0.20857	0.781	-0.421	0.541
			2	-0.36	0.20857	0.123	-0.841	0.121
		2	0	1.11000*	0.20857	0.001	0.629	1.591
			0.5	0.42	0.20857	0.079	-0.061	0.901
			1	0.36	0.20857	0.123	-0.121	0.841
inr	LSD	0	0.5	-.82000*	0.31883	0.033	-1.5552	-0.0848
			1	-1.43000*	0.31883	0.002	-2.1652	-0.6948
			2	-1.15000*	0.31883	0.007	-1.8852	-0.4148
		0.5	0	.82000*	0.31883	0.033	0.0848	1.5552
			1	-0.61	0.31883	0.092	-1.3452	0.1252
			2	-0.33	0.31883	0.331	-1.0652	0.4052
		1	0	1.43000*	0.31883	0.002	0.6948	2.1652
			0.5	0.61	0.31883	0.092	-0.1252	1.3452
			2	0.28	0.31883	0.405	-0.4552	1.0152

		2	0	1.15000*	0.31883	0.007	0.4148	1.8852
			0.5	0.33	0.31883	0.331	-0.4052	1.0652
			1	-0.28	0.31883	0.405	-1.0152	0.4552
pdk	LSD	0	0.5	-0.61	0.28431	0.064	-1.2656	0.0456
			1	-0.11	0.28431	0.709	-0.7656	0.5456
			2	-.90000*	0.28431	0.013	-1.5556	-0.2444
		0.5	0	0.61	0.28431	0.064	-0.0456	1.2656
			1	0.5	0.28431	0.117	-0.1556	1.1556
			2	-0.29	0.28431	0.338	-0.9456	0.3656
		1	0	0.11	0.28431	0.709	-0.5456	0.7656
			0.5	-0.5	0.28431	0.117	-1.1556	0.1556
			2	-.79000*	0.28431	0.024	-1.4456	-0.1344
		2	0	.90000*	0.28431	0.013	0.2444	1.5556
			0.5	0.29	0.28431	0.338	-0.3656	0.9456
			1	.79000*	0.28431	0.024	0.1344	1.4456
akt	LSD	0	0.5	-0.23	0.23527	0.357	-0.7725	0.3125
			1	-0.4	0.23527	0.128	-0.9425	0.1425
			2	-.86000*	0.23527	0.006	-1.4025	-0.3175
		0.5	0	0.23	0.23527	0.357	-0.3125	0.7725
			1	-0.17	0.23527	0.491	-0.7125	0.3725
			2	-.63000*	0.23527	0.028	-1.1725	-0.0875
		1	0	0.4	0.23527	0.128	-0.1425	0.9425
			0.5	0.17	0.23527	0.491	-0.3725	0.7125
			2	-0.46	0.23527	0.086	-1.0025	0.0825
		2	0	.86000*	0.23527	0.006	0.3175	1.4025
			0.5	.63000*	0.23527	0.028	0.0875	1.1725
			1	0.46	0.23527	0.086	-0.0825	1.0025
vg	LSD	0	0.5	-.66000*	0.19035	0.008	-1.0989	-0.2211
			1	-.64000*	0.19035	0.01	-1.0789	-0.2011
			2	-.96000*	0.19035	0.001	-1.3989	-0.5211
		0.5	0	.66000*	0.19035	0.008	0.2211	1.0989
			1	0.02	0.19035	0.919	-0.4189	0.4589
			2	-0.3	0.19035	0.154	-0.7389	0.1389
		1	0	.64000*	0.19035	0.01	0.2011	1.0789
			0.5	-0.02	0.19035	0.919	-0.4589	0.4189
			2	-0.32	0.19035	0.131	-0.7589	0.1189
		2	0	.96000*	0.19035	0.001	0.5211	1.3989
			0.5	0.3	0.19035	0.154	-0.1389	0.7389
			1	0.32	0.19035	0.131	-0.1189	0.7589

* The mean difference is significant at the 0.05 level.

Table S27. ANOVA results of gene expression levels of *ecr*, *kr-h1*, *foxo*, *inr*, *pdk*, *akt*, and *vg* in males.

		**Sum of Squares**	**df**	**Mean Square**	**F**	**Sig.**
ecr	Between Groups	0.309	3	0.103	1.689	0.246
	Within Groups	0.487	8	0.061		
	Total	0.796	11			
kr-h1	Between Groups	0.107	3	0.036	0.29	0.832
	Within Groups	0.989	8	0.124		
	Total	1.096	11			
foxo	Between Groups	0.12	3	0.04	0.647	0.607
	Within Groups	0.493	8	0.062		
	Total	0.613	11			
inr	Between Groups	0.447	3	0.149	4.002	0.052
	Within Groups	0.298	8	0.037		
	Total	0.745	11			
pdk	Between Groups	0.346	3	0.115	3.584	0.066
	Within Groups	0.258	8	0.032		
	Total	0.604	11			
akt	Between Groups	0.296	3	0.099	2.546	0.129
	Within Groups	0.31	8	0.039		
	Total	0.606	11			
vg	Between Groups	0.847	3	0.282	8.399	0.007
	Within Groups	0.269	8	0.034		
	Total	1.116	11			

Table S28. Post hoc tests results of gene expression levels of *ecr*, *kr-h1*, *foxo*, *inr*, *pdk*, *akt*, and *vg* in males.

**Multiple Comparisons**								
**Dependent Variable**		(I) Concentration	(J) Concentration	Mean Difference (I-J)	Std. Error	Sig.	95% Confidence Interval	
							Lower Bound	Upper Bound
ecr	LSD	0	0.5	0.34	0.20154	0.13	-0.1247	0.8047
			1	0.31	0.20154	0.163	-0.1547	0.7747
			2	0.01	0.20154	0.962	-0.4547	0.4747
		0.5	0	-0.34	0.20154	0.13	-0.8047	0.1247
			1	-0.03	0.20154	0.885	-0.4947	0.4347
			2	-0.33	0.20154	0.14	-0.7947	0.1347
		1	0	-0.31	0.20154	0.163	-0.7747	0.1547
			0.5	0.03	0.20154	0.885	-0.4347	0.4947
			2	-0.3	0.20154	0.175	-0.7647	0.1647
		2	0	-0.01	0.20154	0.962	-0.4747	0.4547
			0.5	0.33	0.20154	0.14	-0.1347	0.7947
			1	0.3	0.20154	0.175	-0.1647	0.7647
Kr-h1	LSD	0	0.5	-0.02	0.28708	0.946	-0.682	0.642
			1	-0.23	0.28708	0.446	-0.892	0.432
			2	-0.15	0.28708	0.615	-0.812	0.512
		0.5	0	0.02	0.28708	0.946	-0.642	0.682
			1	-0.21	0.28708	0.485	-0.872	0.452
			2	-0.13	0.28708	0.663	-0.792	0.532
		1	0	0.23	0.28708	0.446	-0.432	0.892
			0.5	0.21	0.28708	0.485	-0.452	0.872
			2	0.08	0.28708	0.788	-0.582	0.742
		2	0	0.15	0.28708	0.615	-0.512	0.812
			0.5	0.13	0.28708	0.663	-0.532	0.792
			1	-0.08	0.28708	0.788	-0.742	0.582
foxo	LSD	0	0.5	0.26	0.20277	0.236	-0.2076	0.7276
			1	0.13	0.20277	0.539	-0.3376	0.5976
			2	0.04	0.20277	0.849	-0.4276	0.5076
		0.5	0	-0.26	0.20277	0.236	-0.7276	0.2076
			1	-0.13	0.20277	0.539	-0.5976	0.3376
			2	-0.22	0.20277	0.31	-0.6876	0.2476
		1	0	-0.13	0.20277	0.539	-0.5976	0.3376
			0.5	0.13	0.20277	0.539	-0.3376	0.5976
			2	-0.09	0.20277	0.669	-0.5576	0.3776
		2	0	-0.04	0.20277	0.849	-0.5076	0.4276
			0.5	0.22	0.20277	0.31	-0.2476	0.6876
			1	0.09	0.20277	0.669	-0.3776	0.5576
inr	LSD	0	0.5	.49000*	0.15759	0.014	0.1266	0.8534
			1	0.14	0.15759	0.4	-0.2234	0.5034
			2	.38000*	0.15759	0.042	0.0166	0.7434
		0.5	0	-.49000*	0.15759	0.014	-0.8534	-0.1266
			1	-0.35	0.15759	0.057	-0.7134	0.0134
			2	-0.11	0.15759	0.505	-0.4734	0.2534
		1	0	-0.14	0.15759	0.4	-0.5034	0.2234
			0.5	0.35	0.15759	0.057	-0.0134	0.7134
			2	0.24	0.15759	0.166	-0.1234	0.6034

		2	0	-.38000*	0.15759	0.042	-0.7434	-0.0166
			0.5	0.11	0.15759	0.505	-0.2534	0.4734
			1	-0.24	0.15759	0.166	-0.6034	0.1234
pdk	LSD	0	0.5	.44000*	0.14657	0.017	0.102	0.778
			1	0.08	0.14657	0.6	-0.258	0.418
			2	0.26	0.14657	0.114	-0.078	0.598
		0.5	0	-.44000*	0.14657	0.017	-0.778	-0.102
			1	-.36000*	0.14657	0.04	-0.698	-0.022
			2	-0.18	0.14657	0.254	-0.518	0.158
		1	0	-0.08	0.14657	0.6	-0.418	0.258
			0.5	.36000*	0.14657	0.04	0.022	0.698
			2	0.18	0.14657	0.254	-0.158	0.518
		2	0	-0.26	0.14657	0.114	-0.598	0.078
			0.5	0.18	0.14657	0.254	-0.158	0.518
			1	-0.18	0.14657	0.254	-0.518	0.158
akt	LSD	0	0.5	.43000*	0.16068	0.028	0.0595	0.8005
			1	0.15	0.16068	0.378	-0.2205	0.5205
			2	0.26	0.16068	0.144	-0.1105	0.6305
		0.5	0	-.43000*	0.16068	0.028	-0.8005	-0.0595
			1	-0.28	0.16068	0.12	-0.6505	0.0905
			2	-0.17	0.16068	0.321	-0.5405	0.2005
		1	0	-0.15	0.16068	0.378	-0.5205	0.2205
			0.5	0.28	0.16068	0.12	-0.0905	0.6505
			2	0.11	0.16068	0.513	-0.2605	0.4805
		2	0	-0.26	0.16068	0.144	-0.6305	0.1105
			0.5	0.17	0.16068	0.321	-0.2005	0.5405
			1	-0.11	0.16068	0.513	-0.4805	0.2605
vg	LSD	0	0.5	.60000*	0.14972	0.004	0.2547	0.9453
			1	-0.02	0.14972	0.897	-0.3653	0.3253
			2	-0.02	0.14972	0.897	-0.3653	0.3253
		0.5	0	-.60000*	0.14972	0.004	-0.9453	-0.2547
			1	-.62000*	0.14972	0.003	-0.9653	-0.2747
			2	-.62000*	0.14972	0.003	-0.9653	-0.2747
		1	0	0.02	0.14972	0.897	-0.3253	0.3653
			0.5	.62000*	0.14972	0.003	0.2747	0.9653
			2	0	0.14972	1	-0.3453	0.3453
		2	0	0.02	0.14972	0.897	-0.3253	0.3653
			0.5	.62000*	0.14972	0.003	0.2747	0.9653
			1	0	0.14972	1	-0.3453	0.3453

* The mean difference is significant at the 0.05 level.
